# Supplementary material for: Moss bugs shed light on the evolution of complex bioacoustic systems
Source: PLoS One. 2024 Feb 23;19(2):e0298174. doi: 10.1371/journal.pone.0298174 (PMC10890781; doi:10.1371/journal.pone.0298174)
Supplement: S1 Table — (DOCX) [file pone.0298174.s001.docx]

**S1 Table.** A list of all the moss bug species examined and their label information.

| **Species** | **Specimen collection site** | **Analysis specimen was used in** |
| --- | --- | --- |
| *Hackeriella brachycephala* | Australia, New South Wales, New England National Park, 03-07.12.2009, sifted from *Dicranoloma dicarpum*, coll. Viktor Hartung | SEM |
| *Hackeriella veitchi* | Australia, Springbrook National Park, summer 2007, from bryophytes, coll. Geoff Monteith | SEM |
| *Hemiodoecus leai* | Australia, New South Wales, Kosciuszko National Park, Rennix Gap, S 36° 21,599’, E 148° 30,482’; elevation 1566 m. 18-27.12.2009, from *Sphagnum cristatum*, coll. Viktor Hartung | SEM, synchrotron micro-CT, photomicrography, confocal laser scanning microscopy |
| *Oiophysa cumberi* | New Zealand, North Island, Tongariro National Park, 16.04.2010, from *Ptychomnion aciculare*, *Leucobryum candidum*, *Echinodium hispidum* and *Camptochaete arbuscula*, coll. Viktor Hartung | SEM |
| *Peloridium hammoniorum* | Chile, Chiloe, Estacion Biologica „Senda Darwin“, Ancud, 02.02.2014, from *Polytrichadelphus magellanus*, coll. Viktor Hartung | SEM, benchtop micro-CT, laser Doppler vibrometry, videography |
| *Xenophyes cascus* | New Zealand, North Island, Orongorongo, Rimutaka forest park, 06.04.2010, from *Dicranoloma dicarpum* ,  *Dicranoloma billarderi*, *Plagiochila stephensoniana* and *Ptychomnion aciculare*, coll. Viktor Hartung; Otaki forks, Tararua forest park, 09-12.04.2010, from unspecified bryophytes and *Racopilum convolutaceum*, colls. Viktor Hartung & George Gibbs; Taranaki National Park, Dawson Falls, 14.04.2010, from *Leucobryum candidum* and *Dicranoloma* sp., coll. Viktor Hartung; Tongariro National Park, 16-17.04.2010, from *Ptychomnion aciculare*, *Leucobryum candidum*, *Echinodium hispidum* and  *Dendrohypopterygium filiculiforme*, coll. Viktor Hartung | SEM, synchrotron micro-CT, confocal laser scanning microscopy |
| *Xenophyes kinlochensis* | New Zealand, South Island, Fiordland National Park, Key Summit track, 06-08.03.2010. from *Chandonanthus squarrosus*, *Plagiochila circinalis*,  *Ptychomnion aciculare*, and  *Sphagnum cristatum*, coll. Viktor Hartung | SEM |
| *Xenophysella stewartensis* | New Zealand, Stewart Island, Horseshoe Bay, 13-17.02.2010, from *Bazzania adnexa*, *Dicranoloma billardieri*, and *Schistochila lehmanniana*, coll. Viktor Hartung | SEM |
