# Supplementary material for: Moss bugs shed light on the evolution of complex bioacoustic systems
Source: PLoS One. 2024 Feb 23;19(2):e0298174. doi: 10.1371/journal.pone.0298174 (PMC10890781; doi:10.1371/journal.pone.0298174)
Supplement: S4 File — (DOCX) [file pone.0298174.s007.docx]

A scanning electron microscopic study led to the establishment of the set of the following 93 characters that were used for phylogenetic analysis of Peloridiidae and their sister groups [1], referred to this manuscript as S1 Supplementary File. The characters are numbered from zero on, since this is the format that is used by the phylogenetic software WinClada [2] that was used for construction of the character matrix. “-“ in the matrix means that the character is inapplicable to the taxon, “?” – that the character state is missing. For the tarsal characters, only the hind legs were considered.

***SEM Characters***

0. Number of tarsal segments: 0 = 2 segments, 1 = 3 segments

1. Tibial spurs: 0 = absent; 1 = present, not forming sockets for setae; 2 = present, forming sockets for setae

Tibiae of many Peloridiidae and other Hemiptera carry what we call here “spurs” - robust cuticular spines without sockets that probably serve better adhesion to the substrate.

2. Tibial spurs formed as sockets for setae: 0 = immovable, 1 = movable

3. Position of tibial spurs: 0 = symmetrical, 1 = asymmetrical

This character is only applicable to the Peloridiidae with 4 tibial spurs (most common number within the family). Two states (in ventral view) are possible: symmetrical arrangement, with one spur laterally on each side and two ventrally (e.g. [1], Supplement 3. Fig. 1. A, B) – or asymmetrical one, with one inner lateral, one ventral and two outer lateral spurs (e.g. [1], Supplement 3. Fig. 1. F, G).

4. Spurs on tarsal segments: 0 = absent, 1 = present

5. Setae on tarsus: 0 = not arranged in rows, 1 = arranged in rows

6. Setae on tarsus: 0 = fluted, 1 = smooth

7. Form of tarsus I: tapered = 0, rounded = 1

The character is applicable only to Peloridiidae

8. Ventral brush: 0 = absent, 1 = present

The apex of the distal tarsal segment in *Pyrrhocoris apterus* (Linnaeus, 1758) [1] carries a row of microtrichia (termed here “ventral brush” after [3, 4]).

9. Ventral flap: 0 = absent; 1 = present, membranous; 2 = present, sclerotized

The ventral distal margin of the last tarsal segment in *Corythucha ciliata* (Say, 1832) [1] is elevated in a sclerotized flap covering the basal region of the unguitractor. In cicadomorphan species studied in [1], the ventral distant margin of the last tarsal segment forms a membranous flap that is stretched towards the unguitractor.

10. Arolium: 0 = absent, 1 = present

11. Arolium: 0 = single-lobed, 1 = bilobed

12. Protrusion on arolium: 0 = absent, 1 = present

In *Cercopis sanguinolenta* (Scopoli, 1763) the arolium has protrusions on both sides that are in close contact with the respective claw tip ([1], see also [4])

13. Arolium (fully inflated) in comparison to claws: 0 = small, 1 = large

14. Contact zone on arolium: 0 = absent, 1 = present

Contact zone is the terminal region of the arolium with a specially structured thickened cuticle that plays an important role in attachment to the substrate [1, 5]. It is quite common in Auchenorrhyncha

15. Number of setae on the ventral side of arolium (one side): 0 = 1 setae, 1 = 2 setae, 2 = 3 setae

16. Pulvilli: 0 = absent, 1 = present

17. Form of the scales on the unguitractor: 0 = more or less rounded, 1 = more or less rectangular

18. Scales on unguitractor: 0 = not arranged in rows, 1 = arranged in rows.

*Psylla alni* (Linnaeus, 1758) has only two scales on unguitractor, so this character is not applicable to the species

19. Number of rows of unguitractor scales: 0 = 2 rows, 1 = 3 rows, 2 = 2 rows + microtrichia forming the middle row

20. The number of scales on unguitractor in lateral and median rows: 0 = similar, 1 = different (mostly the number in lateral rows is double that in the median row)

21. Setiform parempodia: 0 = absent, 1 = present

Here, as in [1], setiform sensilla on unguitractor are considered homologues and are referred to as (setiform) parempodia, whereas the socketless acanthae on unguitractor that are known from some Heteroptera such as *Saldula saltatoria* (Linnaeus, 1758) and *Pyrrhocoris apterus*, are termed accessory parempodia and also considered homologues between the species, although this remains to be proven.

22. Accessory parempodia: 0 = absent, 1 = 1 pair, 2 = 2 pairs

S. the comment to the character 21.

23. Claws: 0 = smooth, 1 = sculptured with microtrichia, 2 = carry setae

24. Basal tooth on the claw: 0 = absent, 1 = present

25. Claws: 0 = not serrated, 1 = serrated

26. Microtrichia between claws and unguitractor: 0 = absent, 1 = present

27. Ventral margin of the antennal flagellum in caudal view: 0 = flat or almost flat, 1 = simply convex; 2 = convex, but with an offset tip being almost flat (s. [1] Supplement 3. figs. 5-6)

Characters 78-30 refer to the very particular antennal structure of Peloridiidae and are not applicable to other taxa. For details and illustrations see [1].

28. Terminal placoid sensillum of the antenna bordered by the furrow and coeloconic sensilla arranged in a more or less straight row = 0; not bordered by the furrow, coeloconic sensilla not in a row = 1

29. Scales on the flagellar petiolus: 0 = slender/weak, not touching each other; 1 = broad, touching each other; 2 = from slender to broad, variable

30. Scales on the fusiform part of the flagellum (ventral view): 0 = not extending into the apical third; 1 = extending into the apical third of the flagellum

31. Flagellum base: 0 = broad, 1 = petiolate

32. Sensilla numbers on the antenna: 0 = low, 1 = high

Low numbers = couple of dozens at most, high = a hundred or more.

33. Campaniform sensilla on pedicel: 0 = absent, 1 = present

34. Olfactory placoid sensilla: 0 = absent, 1 = present

35. Coeloconic sensilla: 0 = absent, 1 = present

36. Genal area: 0 = concave, 1 = flat

Characters 36-40 refer to the highly derived head structure of Peloridiidae and are not applicable to other taxa. For details and illustrations see [1].

37. Genal area medially: 0 = covered with microtrichia, 1 = bare of microtrichia

38. Punctation on genal area: 0 = absent, 1 = present

39. Wax covering on genal area: 0 = absent, 1 = present

40. Hind margin of genal area: 0 = not convex, 1 = convex and reflexed, embracing somewhat the antenna

41. First abdominal tergite: 0 = long and narrow; 1 = short and thick

Characters 41-45 refer to the very particular structure of the dorsal abdomen with plastron-bearing structures that is typical for Peloridiidae and is not applied to other Hemiptera. The width is measured as the longest distance between the arms of posterolateral cuticular apodemes on tergite 1. ([1], Supplement 3., figs. 11-14)

42. Plastron: 0 = present on the whole of abdominal dorsum, 1 = lateral regions of anterior segment borders plastron-free, 2 = lateral regions of posterior segment borders plastron-free

43. Plastron-building microtrichia: 0 = small (2-3 µm), 1 = large (> 10 µm)

44. Microtrichia arrangements: 0 = single, 1 = single, in rows; 2 = single, but arranged in groups of several microtrichia that are aligned in rows; 3 = grouped, originating from a common base

45. Microtrichia on lateral regions of abdominal tergites: 0 = unorganized, 1 = tend to arrange in circles (cell borders?)

46. Microtrichia on abdominal tergites: 0 = sparse, 1 = dense

47. Microtrichia on abdominal tergites: 0 = peg- or knob-like, 1 = hair-like

48. Abdominal tergites: 0 = not covered with wax, 1 = covered with wax

49. Ventral surface of the tegmina: 0 = mostly covered by cuticular sculpture; 1 = sculptural elements are sparse, limited to a very small part of the surface or completely absent

Characters 49-58 refer to the very particular tegminal structure of Peloridiidae and are not applicable to other taxa. For details and illustrations see [1].

50. Ventral sculpture on tegmina: 0 = present only or mostly on veins, 1 = present on veins and membranous areas between them

51. Ventral sculpture on tegmina, ScP: 0 = not sculptured, 1 = sculptured

52. Ventral sculpture on tegmina: 0 = present mostly laterodistally of R and M, 1 = present on the most part of the tegmen, not only beyond R and M

53. Ventral sculpture on tegmina: 0 = absent on clavus, 1 = present on clavus

54. Ventral sculpture on tegmina: 0 = M + CuA with reduced sculpture, 1 = M + CuA with normal sculpture

55. Ventral sculpture on tegmina: 0 = CuP with reduced sculpture, 1 = CuP with normal sculpture

56. Ventral sculpture on tegmina: 0 = apical radial cell without a bare spot that is free of sculpture, 1 = apical radial cell has a bare spot

57. Ventral sculpture on tegmina: 0 = the bare spot on the apical radial cell is marginal, 1 = the bare spot of the apical radial cell reaches the center of the cell or even beyond

58. Ventral sculpture on tegmina, between ScA / ScP and/or on costal cells: 0 = sculpture absent, 1 = sculpture present

59. Ventral sculpture on tegmina: 0 = scale-like acanthae absent, 1 = scale-like acanthae present

60. Ventral sculpture on tegmina: 0 = peg-like microtrichia absent, 1 = peg-like microtrichia present

61. Ventral sculpture on tegmina: 0 = hair-like microtrichia absent, 1 = hair-like microtrichia present

1. Ventral sculpture on tegmina: 0 = “compressed scales” absent, 1 = “compressed scales” present

“Compressed scales” are scale-like acanthae of particular form that have been found so far only in Peloridiidae and are depicted in [1], figure 20c.

63. Ventral sculpture on tegmina (except C, ScP and costal cells): 0 = trichoid sensilla absent, 1 = trichoid sensilla present

64. Ventral sculpture on tegmina: 0 = sculpture on veins same as on membranes, 1 = sculpture on veins different to that on membranes

Character 64 refers to the very particular tegminal structure of Peloridiidae and is not applicable to other taxa. For details and illustrations see [1].

65. Dorsal sculpture on tegmina: 0 = microtrichia absent, 1 = microtrichia present

66. Dorsal sculpture on tegmina: 0 = microtrichia single or at most grouped by 2 or 3, quite sparse; 1 = microtrichia are assembled in larger groups, quite dense

Character 66 refers to the very particular tegminal structure of Peloridiidae and is not applicable to other taxa. For details and illustrations see [1].

67. Dorsal sculpture on tegmina: 0 = trichoid sensilla absent, 1 = trichoid sensilla present on veins, 2 = trichoid sensilla present on most of the tegmen surface

68. Dorsal sculpture on tegmina: 0 = punctation dorsally widespread, 1 = punctation limited to the R stem and AP; 2 = punctation is absent

Character 68 refers to the very particular tegminal structure of Peloridiidae and is not applicable to other taxa. For details and illustrations see [1].

69. Integumental glands: 0 = simple glands absent, 1 = simple glands present

“Simple glands” are simple pores in the cuticle without peripheral elements or complicated orifice structures.

70. Integumental glands type III with peripheral elements: 0 = absent, 1 = present

Peripheral elements of integumental glands are peg-like structures surrounding the orifice of the gland.

71. Integumental glands: 0 = peripheral elements not differentiated; 1 = differentiated into inner and outer circle

In the last case the gland orifice is often covered by the inner elements.

72. Integumental glands, orifice: 0 = not sunk sunk-in; 1 = sunk-in

When the orifice of the gland is sunk-in into the cuticle, the inner peripheral elements are mostly sunk-in, too.

73. Integumental glands, inner elements relative size: 0 = definitely smaller than the outer elements; 1 = not clearly smaller

Characters 73-77 refer to the very particular structure of integumental glands that so far has been found only in Peloridiidae and are not applicable to other taxa. For details and illustrations see [1].

74. Integumental glands, outer elements: 0 = without clubbed tip, 1 = with clubbed tip

75. Integumental glands, outer elements: 0 = their number (or the number of undifferentiated peripheral elements) does not vary with body region; 1 = the number varies with it

76. Integumental glands, on head and pronotum: 0 = glands on that regions are similar to those elsewhere on the body; 1 = glands on dorsal side of head and pronotum are set on cuticular elevations; 2 = glands on both dorsal and ventral side of head and pronotum are set on cuticular elevations; 3 = glands on head and pronotum are sunk-in into the cuticula

77. Integumental glands, individual variation: 0 = absent, 1 = present

78. Integumental glands, on abdominal terga: 0 = abdominal terga do not carry integumental glands; 1 = abdominal terga carry them

79. Integumental glands, on abdominal terga: 0 = absent from plastron regions; 1 = occur under plastron

80. Integumental glands, on abdominal terga: 0 = have structure similar to the glands elsewhere on the body; 1 = have structure different from them

81. Labium tip form: 0 = flat, 1 = skewed, 2 = lipped, 3 = sharp

82. Labium tip, antisutural fissure: 0 = absent, 1 = present

When considering the labial tip, the terms “dorsal” or “ventral”, “anterior” or “posterior” can be confusing. For instance, the labium forms a groove that holds the other mouthparts; the groove is open on one side, which is ontogenetically anterior, but when the labium is clapped backwards by the animal as is normally the case when not feeding, the labial groove is on the ventral side. Thus different papers use different terminology that is often confusing. We apply here, as in [1], the term “sutural” to refer to the side of the labium tip where the labial groove is open, and “antisutural” to refer to the opposite side.

In some hemipterans, there is not only the labial groove opening, but also an additional fissure on the antisutural side that is limited to the tip region.

82. Labium tip, apical lobe: 0 = absent, 1 = present

Apical lobe is a small additional third lobe on the labium tip that is located antisuturally and is present in some predatory Heteroptera

84. Labium tip, multi-peg structures: 0 = absent, 1 = present

Multi-peg structures are citucular formations on the tip of inner side of the labium, most likely contacting the stylets when these are protruding. Their function is unclear and homology between different Hemiptera taxa is postulated here as in [1] but is not proven.

85. Labium tip, central antisutural part of the labium: 0 = not elevated, 1 = elevated

86. Labium tip, antisutural group of the sensilla on the margin of the labium orifice: 0 = absent, 1 = present

In Hemiptera, labial sensilla are mostly not very high in number and can be divided in several groups. One of those is termed here as in [1] the “antisutural group” - these are few pairs of sensilla that are located on the antisutural side of the mouth opening and are most likely in contact with stylets when these are protruding.

The inner trichoid sensilla in Peloridiidae are considered homologous to the antisutural group in Fulgoromorpha, since in many Peloridiidae they are located on the antisutural side of the labium orifice. In these representatives the mandibular stylets are not twisted (for details s. [1]), which is most likely a plesiomoprhous condition, when compared with the inner sensilla located laterally, where the mandibular sensilla are twisted.

87. Labium tip, outer circle of sensilla with (at least presumably) predominantly mechanosensitive function: 0 = absent, 1 = present

88. Labium tip, sutural group of mostly (presumably) gustatory sensilla: 0 = absent, 1 = present

89. Labium tip, cuticula carrying sutural group of sensilla: 0 = not sculptured, 1 = sculptured

90. Labium tip, multiporous (presumably olfactory) sensilla: 0 = absent, 1 = present

For Peloridiidae, even in cases when the coeloconic sensillum does seem to have only a terminal pore and not a multiporous wall, this character is coded as “present”, since the sensilla with a single pore are clearly homologous to the multiporous ones in other Peloridiidae.

91. Labium tip, inner trichoid sensilla: 0 = located antisuturally of the labium orifice, mandibular stylets not twisted; 1 = laterally of the labium orifice, mandibular stylets twisted

The characters 91-92 refer to a condition that is present only in Peloridiidae. For details and illustrations s. [1].

92. Labium tip, coeloconic sensilla: 0 = multiporous, 1 = terminal pore, 2 = socketed, with a terminal pore

***Abdominal Characters***

The additional 18 characters of abdominal anatomy were established by an examination of the species. These are included in S2-S3 Supplementary Files.

0. Ridge on thoracic-abdominal boundary: 0 = absent; 1 = present.

All examined Cicadomorpha and Fulgoromorpha [6] and certain Pentatomomorpha (personal observation) possess a ridge which connects the metathorax to the first abdominal segment.

1. Ridge: 0 = without seta on dorsal surface; 1 = with one large seta on its dorsal surface.

The ridge of Fulgoromorpha is always provided with a distinct, long seta on its dorsal surface [6, 7]. This seta has not been observed in Cicadomorpha [6] or Pentatomomorpha (personal observation).

2. Anterior portion of tergum 1: 0 = sclerotised; 1 = membranous.

In most Hemiptera, the abdomen is connected to the thorax by a narrow membrane [6, 8, this study]. However, in some species, the main body of abdominal tergum I may be either a single sclerotized plate, or its anterior portion might be membranous [6-8]. This character is not applicable to the Psylloidea and the Aleyrodoidea, as their first abdominal tergum is modified into a flattened sclerotized plate which is entirely fused to the metathorax [9].

3. Shape of tergum I: 0 = simple; 1 = lobe-like.

Tergum I may have the form of a simple plate, as in most Heteroptera [8, 10], or it may be modified into a lobe-like shape, which is the site of frequency multiplier mechanisms for the generation of vibroacoustic signals [6]. In most Cicadomorpha, the lateral margin of tergum I is modified into a lobe which bears tymbals or tymbal-like organs [6], whereas in Fulgoromorpha, the entirety of tergum I is modified into a lobe-like shape known as the snapping organ [7].

4. Tergum I in cross-section: 0 = strongly convex; 1 = flat.

Strongly convex abdominal terga in cross-section characterise the Auchenorrhyncha and Sternorrhyncha. Flattened terga have been proposed as a synapomorphy uniting Coleorrhyncha and Heteroptera [11]. Uniquely among Heteroptera, certain Aenictopecheidae (Enicocephalomorpha) also have strongly convex terga [8].

5. Terga I-II: 0 = not surrounded by membrane; 1 = surrounded by membrane.

In Auchenorrhyncha and Coleorrhyncha, terga I-II are typically surrounded by membranous cuticle, presumably belonging to both segments, although the segmental boundaries cannot be determined. In Heteroptera, the abdominal segments are usually well-sclerotised, although small forms such as some Dipsocoromorpha (e.g. *Ceratocombus* sp., but not in *Guapinannus* sp.) may be membranous (personal observation). This character is not applicable for Sternorrhyncha, as in the examined Aleyrodoidea and Psylloidea, the base of the abdomen is distinctly constricted into a narrow “waist” [9], which is found nowhere else in Hemiptera.

6. Membranous area between metathorax and tergum I with a plate bearing setae-like structures: 0 = absent; 1 = present.

Ossiannilsson [12] described this plate from Cicadomorpha as a “pilose area”, to which he ascribed a tentative sensory function. Its detailed morphology has remained unstudied.

7. Antecosta of tergum II: 0 = deeply impressed; 1 = faint antecosta.

In Heteroptera, terga I-II are distinctly fused to each other, forming a vibrational organ known as the tergal plate (not to be confused with the synonymous structure found in aradids) [8, 10].

8. Fields of sensory setae on anterior margin of tergum II: 0 = absent; 1 = present.

Such a field was described by [12] as being present in all Delphacidae.

9. Cuticular bridge linking terga I-II: 0 = absent; 1 = present.

10. Spiracle I: 0 = free on membrane of tergum I; 1 = fused to metathorax.

The first abdominal spiracle is fully incorporated onto the metathorax in the Auchenorrhyncha [6, 7], most Heteroptera [8] and the Sternorrhyncha [9]. Exceptions include the soft-bodied Enicocephalomorpha and Dipsocoromorpha, where spiracle 1 is free [8, 13].

11. Spiracle II: 0 = separate from tergum 2, either on a membrane of a sclerite; 1 = fused to tergum 2.

Abdominal spiracle II fused to tergum II in most Hemiptera, with the exception of some Cicadomorpha, where spiracle II is detached from this segment in part and lies on a distinct sclerite, or on the membrane [6]. The ventralised spiracle II of some Heteroptera represents a laterotergal fusion to the sternum [14].

12. Tergum III: 0 = roughly as broad as terga I-II; 1 = much broader than terga I-II, its lateral margin surrounding the latter.

13. Sternum I in adult: 0 = externally visible as an independent sclerite; 1 = extremely reduced, not visible externally, fully incorporated to metathorax, forming a secondary postcoxale.

14. Sterna I-II: 0 = simple; 1 = subdivided.

Auchenorrhyncha are unique in that their sterna I-II are distinctly subdivided [6, 7, 15, 16].

15. Ventral longitudinal muscle of sternum I in adult: 0 = absent; 1 = present.

The ventral longitudinal muscle of abdominal segment I is invariably missing in adult Coleorrhyncha [this study] and Heteroptera [8], but persists in the Auchenorrhyncha [6, 7]. The condition in Sternorrhyncha is complex. Weber [9, 17] observes a single muscle linking the metathorax, which he interpreted as a fusion of the metathoracic ventral longitudinal (IIIvlm2) with the ventral longitudinal muscle of sternum I (vlm1), although what he meant by the term “fusion” is morphologically and developmentally ambiguous. We interpret this muscle as IIIvlm2, and treat vlm1 as absent in adult Sternorrhyncha.

16. Dorsoventral muscles of abdominal segments I-II: 0 = normal; 1 = hypertrophied.

Different pairs of dorsoventral muscles of abdominal segments I-II are hypertrophied in most Cicadomorpha, where they form part of a tymbal or tymbal-like mechanism [6, 12]. Similar hypertrophy takes place in the dorsoventral muscles of most non-Asiracinae Delphacidae [7].

17. Dorsal longitudinal muscles of abdominal segments I-II: 0 = normal; 1 = hypertrophied.

Enlarged dorsal longitudinal muscles of tergum I operate the snapping organ which defines Fulgoromorpha [6, 7].

***References***

1. Hartung V. Systematics of Peloridiidae (Insecta: Hemiptera: Coleorrhyncha) - an integrative approach. PhD Thesis, Humboldt Universität zu Berlin. 2018. Available from: https://doi.org/10.18452/19405

2. Nixon KC. Winclada (BETA) Version 1.00.08. published by the author, Nixon KC. 1999.

3. Weirauch C. Pretarsal structures in Reduviidae (Heteroptera, Insecta). Act Zool. 2005; 86: 91-110.

4. Friedemann K, Spangenberg R, Yoshizawa K, Beutel RG. Evolution of attachment structures in the highly diverse Acercaria (Hexapoda). Cladistics 2014; 30: 170-201.

5. Friedemann K, Beutel RG. Morphology of arolia in Auchenorrhyncha (Insecta, Hemiptera). J Morphol. 2014; 275: 1217-1225.

6. Davranoglou LR, Mortimer B, Taylor GK, Malenovský I. On the morphology and evolution of cicadomorphan tymbal organs. Arthropod Struct Dev. 2020; 55: 100918.

7. Davranoglou LR, Cicirello A, Mortimer B, Taylor GK. Planthopper bugs use a fast, cyclic elastic recoil mechanism for effective vibrational communication at small body size. PLoS Biol. 2019; 17: e3000155.

8. Davranoglou LR, Baňař P, Schlepütz CM, Mortimer B, Taylor GK. The pregenital abdomen of Enicocephalomorpha and morphological evidence for different modes of communication at the dawn of heteropteran evolution. Arthropod Struct Dev. 2017; 46, 843-868.

9. Weber H. Der Bau der Imago der Aleurodinen. Ein Beitrag zur vergleichenden Morphologie des Insektenkörpers. E. Schweizerbartsche Verlagsbuchhandlung (Erwin Nägele), Stuttgart (1935).

10. Gogala M. Vibration producing structures and songs of terrestrial Heteroptera as systematic characters. Biol Vestn. 1984; 32, 19-36.

11. Schlee D. Morphologie und Symbiose, ihre Beweiskraft für die Verwandtschaftsbeziehungen der Coleorrhyncha (Insecta, Hemiptera). Phylogenetische Studien an Hemiptera, IV: Heteropteroidea (Heteroptera & Coleorrhyncha) als monophyletische Gruppe. Stutt Beit Naturk. 1969; 210, 1-27.

12. Ossiannilsson F. Insect drummers, a study on the morphology and function of the sound-producing organ of Swedish Homoptera Auchenorrhyncha. Opus Entomol. 1949; Suppl. X: 1-145.

13. Štys P. A new family of Heteroptera with dipsocoromorphan affinities from Papua New Guinea. Acta Entomol Bohemoslov. 1983; 80: 256-292.

14. Sweet MH. Comparative external morphology of the pregenital abdomen of the hemiptera C.W. Schaefer (Ed.), Studies on Hemipteran Phylogeny, Thomas Say Publications in Entomology: Proceedings, Entomol Soc Am Lanham; 1996; pp. 119-158.

15. Kramer S. Morphology and phylogeny of auchenorrhynchous hemiptera (Insecta). Ill Biol Monogr 1950; 20:1-111.

16. Snodgrass RE. Morphology of the insect abdomen. Part II. The genital ducts and the ovipositor. Smithsonian Misc Collect, 89 (1933), pp. 1-148.

17. Weber H. Biologie der Hemipteren. Biolog Stud, Bonn; 1930;, p. 543.
