# Supplementary material for: Moss bugs shed light on the evolution of complex bioacoustic systems
Source: PLoS One. 2024 Feb 23;19(2):e0298174. doi: 10.1371/journal.pone.0298174 (PMC10890781; doi:10.1371/journal.pone.0298174)
Supplement: Attachment — Submitted filename: PONE-D-23-39772.pdf [file pone.0298174.s008.pdf]

PLOS ONE

Moss bugs shed light on the evolution of complex bioacoustic systems

--Manuscript Draft--

**Manuscript Number:**	PONE-D-23-39772
**Article Type:**	Research Article
**Full Title:**	Moss bugs shed light on the evolution of complex bioacoustic systems
**Short Title:**	Moss bug bioacoustic evolution.
**Corresponding Author:**	Leonidas-Romanos Davranoglou, DPhil<br>Oxford University Museum of Natural History<br>Oxford, Oxfordshire UNITED KINGDOM
**Keywords:**	
**Abstract:**	Vibroacoustic signalling is one of the dominant strategies of animal communication, especially in small invertebrates. Among insects, the order Hemiptera displays a staggering diversity of vibroacoustic organs and is renowned for possessing biomechanically complex elastic recoil devices such as tymbals and snapping organs that enable robust vibrational communication. However, our understanding of the evolution of hemipteran elastic recoil devices is hindered by the absence of relevant data in the phylogenetically important group known as moss bugs (Coleorrhyncha), which produce substrate-borne vibrations through an unknown mechanism. In the present work, we reveal the functional morphology of the moss bug vibrational mechanism and study its presence across Coleorrhyncha and in extinct fossilised relatives. We incorporate the anatomical features of the moss bug vibrational mechanism in a phylogeny of Hemiptera, which supports either a sister-group relationship to Heteroptera, or a sister-group relationship with the Auchenorrhyncha. Regardless of topology, we propose that simple abdominal vibration was present at the root of derived Hemiptera, and arose 350 million years ago, suggesting that this mode of signalling is among the most ancient in the animal kingdom. Therefore, the most parsimonious explanation for the origins of complex elastic recoil devices is that they represent secondary developments that arose exclusively in the Auchenorrhyncha.
**Order of Authors:**	Leonidas-Romanos Davranoglou, DPhil
	Viktor Hartung, PhD
**Additional Information:**	
**Question**	**Response**
**Financial Disclosure**<br><br>Enter a financial disclosure statement that describes the sources of funding for the work included in this submission. Review the submission guidelines for detailed requirements. View published research articles from *PLOS ONE* for specific examples.<br><br>This statement is required for submission and **will appear in the published article** if the submission is accepted. Please make sure it is accurate.	Yes

Powered by Editorial Manager® and ProduXion Manager® from Aries Systems Corporation

**Funded studies**<br>Enter a statement with the following details:<br>• Initials of the authors who received each award<br>• Grant numbers awarded to each author<br>• The full name of each funder<br>• URL of each funder website<br>• Did the sponsors or funders play any role in the study design, data collection and analysis, decision to publish, or preparation of the manuscript?<br><br>Did you receive funding for this work?	
Please add funding details.<br>as follow-up to "**Financial Disclosure**<br><br>Enter a financial disclosure statement that describes the sources of funding for the work included in this submission. Review the submission guidelines for detailed requirements. View published research articles from *PLOS ONE* for specific examples.<br><br>This statement is required for submission and **will appear in the published article** if the submission is accepted. Please make sure it is accurate.<br><br>**Funded studies**<br>Enter a statement with the following details:<br>• Initials of the authors who received each award<br>• Grant numbers awarded to each author<br>• The full name of each funder<br>• URL of each funder website<br>• Did the sponsors or funders play any role in the study design, data collection and analysis, decision to publish, or preparation of the manuscript?<br><br>Did you receive funding for this work?"	Leverhulme Trust Early Career Fellowship grant (ECF-2021-199); Elsa Neuman doctorate grant (application number H49023); German Academic Exchange travel grant (processing number D/09/04219)
Please select the country of your main research funder (please select carefully as in some cases this is used in fee calculation).	UNITED KINGDOM - GB

as follow-up to "**Financial Disclosure**<br><br>Enter a financial disclosure statement that describes the sources of funding for the work included in this submission. Review the submission guidelines for detailed requirements. View published research articles from *PLOS ONE* for specific examples.<br><br>This statement is required for submission and **will appear in the published article** if the submission is accepted. Please make sure it is accurate.<br><br>**Funded studies**<br>Enter a statement with the following details:<br>• Initials of the authors who received each award<br>• Grant numbers awarded to each author<br>• The full name of each funder<br>• URL of each funder website<br>• Did the sponsors or funders play any role in the study design, data collection and analysis, decision to publish, or preparation of the manuscript?<br><br>Did you receive funding for this work?"	
**Competing Interests**<br><br>Use the instructions below to enter a competing interest statement for this submission. On behalf of all authors, disclose any competing interests that could be perceived to bias this work—acknowledging all financial support and any other relevant financial or non-financial competing interests.<br><br>This statement is required for submission and **will appear in the published article** if the submission is accepted. Please make sure it is accurate and that any funding sources listed in your Funding Information later in the submission form are also declared in your Financial Disclosure statement.	The authors have declared that no competing interests exist.

View published research articles from *PLOS ONE* for specific examples.<br><br>**NO authors have competing interests**<br><br>Enter: *The authors have declared that no competing interests exist.*<br><br>**Authors with competing interests**<br><br>Enter competing interest details beginning with this statement:<br><br>*I have read the journal's policy and the authors of this manuscript have the following competing interests: [insert competing interests here]*<br><br>* typeset	
**Ethics Statement**<br><br>Enter an ethics statement for this submission. This statement is required if the study involved:<br><br>• Human participants<br>• Human specimens or tissue<br>• Vertebrate animals or cephalopods<br>• Vertebrate embryos or tissues<br>• Field research<br><br>Write "N/A" if the submission does not require an ethics statement.<br><br>General guidance is provided below. Consult the submission guidelines for detailed instructions. **Make sure that all information entered here is included in the Methods section of the manuscript.**	Field research in Australia was undertaken under permits № WITK06355209 (Queensland), S13005 (New South Wales), 10005138 (Victoria) and FA10018 (Tasmania); in New Zealand under WE-26346-RES; in Chile under a permit issued by CONAF.

Powered by Editorial Manager® and ProduXion Manager® from Aries Systems Corporation

**Format for specific study types**<br><br>**Human Subject Research (involving human participants and/or tissue)**<br>• Give the name of the institutional review board or ethics committee that approved the study<br>• Include the approval number and/or a statement indicating approval of this research<br>• Indicate the form of consent obtained (written/oral) or the reason that consent was not obtained (e.g. the data were analyzed anonymously)<br><br>**Animal Research (involving vertebrate animals, embryos or tissues)**<br>• Provide the name of the Institutional Animal Care and Use Committee (IACUC) or other relevant ethics board that reviewed the study protocol, and indicate whether they approved this research or granted a formal waiver of ethical approval<br>• Include an approval number if one was obtained<br>• If the study involved *non-human primates*, add *additional details* about animal welfare and steps taken to ameliorate suffering<br>• If anesthesia, euthanasia, or any kind of animal sacrifice is part of the study, include briefly which substances and/or methods were applied<br><br>**Field Research**<br><br>Include the following details if this study involves the collection of plant, animal, or other materials from a natural setting:<br>• Field permit number<br>• Name of the institution or relevant body that granted permission	
**Data Availability**<br><br>Authors are required to make all data underlying the findings described fully available, without restriction, and from the time of publication. PLOS allows rare exceptions to address legal and ethical concerns. See the PLOS Data Policy and FAQ for detailed information.	Yes - all data are fully available without restriction

A Data Availability Statement describing where the data can be found is required at submission. Your answers to this question constitute the Data Availability Statement and **will be published in the article**, if accepted.<br><br>**Important:** Stating 'data available on request from the author' is not sufficient. If your data are only available upon request, select 'No' for the first question and explain your exceptional situation in the text box.<br><br>Do the authors confirm that all data underlying the findings described in their manuscript are fully available without restriction?	
**Describe where the data may be found in full sentences. If you are copying our sample text, replace any instances of XXX with the appropriate details.**<br><br>• If the data are **held or will be held in a public repository**, include URLs, accession numbers or DOIs. If this information will only be available after acceptance, indicate this by ticking the box below. For example: *All XXX files are available from the XXX database (accession number(s) XXX, XXX.*).<br>• If the data are all contained **within the manuscript and/or Supporting Information files**, enter the following: *All relevant data are within the manuscript and its Supporting Information files.*<br>• If neither of these applies but you are able to provide **details of access elsewhere**, with or without limitations, please do so. For example:<br><br>*Data cannot be shared publicly because of [XXX]. Data are available from the XXX Institutional Data Access / Ethics Committee (contact via XXX) for researchers who meet the criteria for access to confidential data.*<br><br>*The data underlying the results presented in the study are available from (include the name of the third party*	All evidence used to support our conclusions is provided within the manuscript. Full tomographic data cannot be shared publicly because they are embargoed until 2026 when our research on them will be finalised. We will then submit them to online databases, such as CXIDB.

Powered by Editorial Manager® and ProduXion Manager® from Aries Systems Corporation

and contact information or URL).<br>• This text is appropriate if the data are owned by a third party and authors do not have permission to share the data.<br><br>* typeset	
Additional data availability information:	Tick here if the URLs/accession numbers/DOIs will be available only after acceptance of the manuscript for publication so that we can ensure their inclusion before publication.

Powered by Editorial Manager® and ProduXion Manager® from Aries Systems Corporation

Manuscript Click here to access/download;Manuscript;Manuscript.pdf

Moss bugs shed light on the evolution of complex bioacoustic systems

Leonidas-Romanos Davranoglou[1*] & Viktor Hartung[2, 3]

[1]Oxford University Museum of Natural History, Parks Road, University of Oxford, Oxford, OX1 3PW, UK

* leonidas-romanos.davranoglou@oum.ox.ac.uk

[2]LWL-Museum of Natural History, Westphalian State Museum with Planetarium, Sentruper Str. 285, 48161 Münster, Germany

[3]Museum für Naturkunde - Leibniz Institute for Evolution and Biodiversity Science, Invalidenstr. 43, 10115 Berlin, Germany

Abstract

Vibroacoustic signalling is one of the dominant strategies of animal communication, especially in small invertebrates. Among insects, the order Hemiptera displays a staggering diversity of vibroacoustic organs and is renowned for possessing biomechanically complex elastic recoil devices such as tymbals and snapping organs that enable robust vibrational communication. However, our understanding of the evolution of hemipteran elastic recoil devices is hindered by the absence of relevant data in the phylogenetically important group known as moss bugs (Coleorrhyncha), which produce substrate-borne vibrations through an unknown mechanism. In the present work, we reveal the functional morphology of the moss bug vibrational mechanism and study its presence across Coleorrhyncha and in extinct fossilised relatives. We incorporate the anatomical features of the moss bug vibrational mechanism in a phylogeny of Hemiptera, which supports either a sister-group relationship to Heteroptera, or a sister-group relationship with the Auchenorrhyncha. Regardless of topology, we propose that simple abdominal vibration was present at the root of derived Hemiptera, and arose 350 million years

ago, suggesting that this mode of signalling is among the most ancient in the animal kingdom. Therefore, the most parsimonious explanation for the origins of complex elastic recoil devices is that they represent secondary developments that arose exclusively in the Auchenorrhyncha.

Key Words: vibrational communication, tremulation, elastic recoil devices, Coleorrhyncha, Hoploridiidae, Peloridiidae, Karabasiidae, Progonocimicidae, Hemiptera

Introduction

Communication using substrate-borne vibrations is one of the dominant signalling modalities among animals, being used by at least 200,000 species [1–3]. Vibrational signalling is particularly widespread in small invertebrates, especially insects, which they use in an array of behavioural contexts, such as courtship [4–6], competition [7, 8], the transmission of alarm and defensive signals [9-11], and even to trigger synchronous egg-hatching [12]. Among them, true bugs (Hemiptera), a megadiverse insect group with over 107.000 described species [13-15], display the greatest diversity of vibroacoustic organs in the animal kingdom [16]. Stink bugs and their allies (Heteroptera) use abdominal vibration (tremulation) [3], tapping [17, 18], wing buzzing [19], and stridulation [16] to generate simple substrate borne vibrations. Conversely, various groups of the suborder Auchenorrhyncha employ specialised elastic recoil devices to generate complex vibroacoustic signals that would be impossible to achieve with muscle action alone. The most widely used elastic recoil devices in Auchenorrhyncha involve the buckling of drum-like tymbals in cicadas and their relatives (Cicadomorpha) [20, 21] and the recently discovered snapping organs in planthoppers (Fulgoromorpha) [22]. Unsurprisingly, the evolutionary origins of complex traits such as hemipteran vibroacoustic elastic recoil mechanisms have remained a matter of debate [21-24]. Their intricate and microscopic morphologies render them challenging to study, their extreme diversity makes homologizing structures between taxa very difficult, their vibroacoustic organs rarely fossilise, and the

systematic placement of certain groups has remained inconclusive, thereby hindering our understanding of how hemipteran biomechanics has changed across time. An elusive group of hemipterans known as the moss bugs (Coleorrhyncha: Peloridiidae) may offer additional insights on the character acquisition that led to the diversity of Hemipteran vibroacoustic mechanisms that we observe today.

Coleorrhyncha (Fig. 1) are a relict group that comprises a single recent family, the Peloridiidae, with 37 small (normally 2-3 mm), cryptically coloured, mostly wingless species that feed on bryophytes, and are rarely collected [25-27].

Figure 1. Two males of *Hackeriella veitchi*, showing the habitus of a generalised moss bug. (photograph courtesy of Jürgen Deckert).

While Peloridiidae are characterised by a Gondwanan distribution, being found in Australia, Lord Howe Island, New Caledonia, New Zealand and southern South America [26-30], their putative extinct relatives that date from the Permian to Cretaceous periods, have been found in Argentina, Australia and Eurasia (31-40). Coleorrhyncha are characterised by the following suite of unique adaptations: to cope with their deficient diet, Peloridiidae possess biosynthetic bacterial endosymbionts that cover their nutrient requirements [41, 42]; their body surface supports a plastron that allows them to stay submerged underwater, a useful trait when living in moist bryophytes [43]; and they perform high-speed jumps (1.5 m s^{-1}) to evade predators [44]. At least one species of Peloridiidae, *Hackeriella veitchi* (Hacker, 1932), has been shown to communicate with low frequency (82 Hz) vibrational signals, which are thought to be produced by a tymbal mechanism [45], although this has not been confirmed experimentally. Indeed, the morphology and biomechanics of coleorrhynchan vibrational organs have remained poorly known, which is a major gap in our understanding of the evolution of hemipteran vibrational organs. The most intriguing question here is whether Peloridiidae (and

Coleorrhyncha) use complex elastic recoil devices or simple abdominal vibration. Furthermore, the behavioural context of the emitted signals is not known [45].

The phylogenetic placement of moss bugs to other Euhemiptera (= Auchenorrhyncha + Coleorrhyncha + Heteroptera) [46-48] (Fig. 2) has remained controversial. Some morphological and molecular studies support Coleorrhyncha as the sister group to Heteroptera, jointly forming the suborder known as the Heteropterodea (Fig. 2A) [46, 47, 49-51], while others recover Coleorrhyncha as sister to Auchenorrhyncha (Fig. 2B), rendering Heteropterodea paraphyletic [52-54]. Based on the above, the elusive relationships of Coleorrhyncha, coupled with the unknown morphology and biomechanics of their vibrational mechanism, prevent us from understanding how complex character states, such the elastic recoil devices that define Auchenorrhyncha, evolved from simpler precursors.

Figure 2. Some of the previous topologies of the moss bug (Coleorrhyncha) position on hemipteran phylogeny. A) A sister-group relationship to Heteroptera (forming the lineage Heteropterodea), as suggested by (37, 38, 40–42); B) alternative topology, where Coleorrhyncha represent the sister-group to Auchenorrhyncha [based on (43–45)].

To address this knowledge gap, we use state-of-the-art synchrotron X-ray microtomography, scanning electron microscopy, and laser Doppler vibrometry to document the morphology and preliminary biomechanics of coleorrhynchan vibrational organs. Through the examination of fossil Coleorrhyncha, we present indications of possible morphological conservatism in moss bug vibrational organs, which may have remained largely unchanged at least since the Late Jurassic (160 mya). To reconstruct the evolution of vibroacoustic mechanisms of Euhemiptera, we integrate the newly acquired morphological data into a morphological matrix comprising 111 characters. We show that complex elastic recoil devices evolved at the root of Auchenorrhyncha, whereas simple abdominal vibration likely represents the plesiomorphic state in Euhemiptera.

Results

Morphology of the moss bug pregenital abdomen

To characterise the morphology of the coleorrhynchan putative abdominal vibrational organ, we examined the pregenital abdomen of five species, covering a considerable proportion of peloridiid diversity (S1 Table). The following description is based on the combined observations on all these species. As in other Euhemiptera, the putative vibrational organ spans the first two abdominal segments [21, 22], which are largely membranous to confer flexibility, except for two central sclerotised plates formed by tergites one and two, respectively (Fig. 3A-B, D). Externally visible elastic recoil devices such as a tymbal or a snapping organ are not present.

Figure 3. External morphology of the moss bug pregenital abdomen. including the vibrational organ, as visualised by confocal laser scanning microscopy. A) Dorsal view of thorax and proximal pregenital abdomen of *Hemiodoecus leai*; B) same, magnified view; C) ventral view of thorax and pregenital abdomen of *H. leai*; D) dorsal view of proximal pregenital abdomen of *Xenophyes cascus*. Abbreviations: crn = median carina; dvms = dorsoventral muscles; ips = intrapostnotal suture; ltg = laterotergite; PLR = posterolateral ridge; pnp = posterior notal wing process; pnt = postnotum; scl = scutellum; scm = scutum; set = setae; sp = spiracle; sss = scuto-scutellar suture; st = sternite; t = tergite.

Tergite one is long but narrow in most peloridiids from Australia and South America (Figs. 3A-B, D, 4A-C, 5A-B), whereas in certain genera (*Oiophysa*, *Xenophyes*) from New Zealand it is short and broad (Figs. 4D-F, 6A). The proportions of the first abdominal tergite may be phylogenetically and biomechanically informative. In all Peloridiidae examined, a distinct posterolateral ridge (PLR) is present on tergite one, whose shape is species-specific (Fig. 4).

Figure 4. Scanning electron microscopy images of the dorsal abdomen of a range of moss bugs, showing the morphological uniformity of the putative vibrational mechanism. A) *Hackeriella brachycephala*; B) *Hemiodoecus leai*; C) *Peloridium hammoniorum*; D) *Oiophysa cumberi*; E)

Xenophyes kinlochensis; F) *Xenophysella stewartensis*. The white arrow indicates the position of the posterolateral ridge.

Figure 5. The moss bug vibrational organ in different sexes and life stages of the species *Hemiodoecus leai*, as visualised by a three dimensional (3D) volumetric reconstruction of synchrotron X-ray microtomography images. A) Male, dorsal view; B) female, dorsal view; C) last instar nymph, dorsal view; D) same, ventral view. Abbreviations: PLR = posterolateral ridge; sp = spiracle;st = sternite; t = tergite.

Figure 6. Three dimensional (3D) volumetric reconstruction of exoskeleton and part of the musculature of the vibrational organ of the moss bug *Xenophyes cascus*, based on images generated by synchrotron X-ray microtomography. A) Dorsal view; B) same, showing the dorsal longitudinal muscles (dlms); C) ventral view of pregenital abdomen, with an emphasis on the robust metafurca (fu3, in yellow); D) same, showing the ventral longitudinal muscles (vlms), focussing on the enlarged IIIvlm2 muscle that attaches on the metafurca; E) ventral view of proximal pregenital abdomen, showing the presence of a ring-like sternite one (stn1); F) same, showing the ventral longitudinal musculature of the region. Abbreviations: dlm = dorsal longitudinal muscle; pnt = postnotum ; sp = spiracle; t = tergite; stn = sternite; vlm = ventral longitudinal muscle.

The form and arrangement of the posterolateral ridge co-varies with the dimensions of the first tergite: in longer and narrower tergites there are two lateral folds that join the posterior apodeme in an obtuse angle (Figs. 3A-B, D, 4A-C, 5A-B), whereas broader and shorter tergites possess a posterolateral ridge that is confined only to the posterior margin of tergite one, bordering the antecosta (Figs. 4D-F, 6A). Spiracle one is placed on a semilunar-shaped sclerite that is fused to the metapostnotum (Fig. 3B). Tergite two is reduced to a narrow strip in all Coleorrhyncha examined (Figs. 3A-B, D; 4; 5A-B) and exhibits no significant variation. Spiracle two is always located on the anterolateral margin of tergite two (Figs. 3A-B, D; 4; 5A-B, 6A), a condition similar to that of Auchenorrhyncha [21, 22]. All remaining spiracles are located ventrally on the connexivum (laterotergites) (Figs. 3C, 5D, 6C). Although mentioned

by previous studies as absent (30), a ring-like sternite one was found in all species studied (Figs. 3C, 6C, E). Sternite one is absent from most Heteroptera apart from some Enicocephalomorpha [55] and Gerromorpha [56] (as a secondary reversal) but is present in all Auchenorrhyncha [21, 22]. The posterior margin of sternite one is provided with a pair of apodemes which act as ~~the~~ attachment sites for muscles (Fig. 7A, apo 1). Sternite two is strongly constricted medially and expanded laterally (Fig. 7A). The border between sternites two and three bears a pair of very large apodemes (Fig. 7A, apo 2), which have been previously interpreted as part of a tymbal mechanism [23]. Similar apodemes are present in Auchenorrhyncha [20-23], although their homology to those of the Coleorrhyncha is unclear. The remaining pregenital tergites and sternites are morphologically unspecialised. A distinct carina is present on sternites three to five (Fig. 3C, crn).

Figure 7. Reconstruction of skeletonmuscular system of the moss bug vibrational organ. A) Schematic illustration of the skeletonmusculature of a generalized moss bug (based on sections and X-ray microtomography of several species); B) dorsal musculature of *Peloridium hammoniorum*, 3D volumetric reconstruction of benchtop X-ray microtomography images. White dashed lines indicate the outline of muscles that are not preserved in this particular specimen, but were observed in sections of this species, and in X-ray images of related species. The outline of the posterolateral ridge was traced from the external surface of the 3D volumetric reconstruction. Abbreviations: apo = apodeme; dlm = dorsal longitudinal muscle; dvm = dorsoventral muscle; plr = posterolateral ridge; stn = sternite; t = tergite; vlm = ventral longitudinal muscle.

Eleven pairs of muscles are directly associated with the moss bug vibrational mechanism (Fig. 7), comprising two pairs of dorsal longitudinal muscles (DLMs), two pairs of ventral longitudinal muscles (VLMs), and six pairs of dorsoventral muscles (DVMs) (for a list of the origins and attachments of pregenital musculature, refer to S2 Table). Unlike the muscles that operate the elastic recoil devices of other Hemiptera [20-23], none of the moss bug pregenital

muscles display hypertrophy that would enable high power motions (Fig. 7A). The largest muscles are the sheetlike VLMs (Figs. 6D, 7A) and DLMs (Figs. 6B, 7B). None of the muscles attach to the PLR (Figs. 6B, 7B). The simple musculature of the moss bug mechanism is more similar to the pregenital abdomen of many Heteroptera [55, 57], which produce vibrational signals by means of simple abdominal tremulation [16, 58].

The peloridiid pregenital abdomen does not display any noticeable sexual dimorphism (Fig. 5), with only tergite 1 being slightly broader and with a thicker posterolateral ridge in females (Fig. 5B). Acoustic signals have so far only been recorded from males (Hartung, in prep.). The moss bug vibrational organ is probably functional only in the adult stage, as in nymphs, the first two abdominal pregenital segments are undeveloped, largely fused and probably immobile, both dorsally and ventrally (Fig. 5C-D). Also, nymphs of several species were investigated via laser vibrometry in the same way that adults were, and did not produce any signals.

Bioacoustics and biomechanics of the moss bug vibrational mechanism

Records of *Peloridium hammoniorum* Breddin, 1897, *Peloridium pomponorum* Shcherbakov, 2014 and *Xenophyes cascus* Bergroth, 1924 were produced with laser vibrometry. For the two *Peloridium*, video recordings of singing males could also be made (S1 video). The features of the songs and generalized information on Peloridiidae are to be summarized elsewhere (Hartung, in preparation); here we use the signal structure of *P. hammoniorum* (whose movements while singing are identical to *P. pomponorum*), our best studied species, to extract information on the morphology of the vibroacoustic apparatus.

The call of *Peloridium hammoniorum* is a simple monotonous sequence of pulses with a fundamental frequency of approximately 500-600 Hz, and a dominant frequency extending to 4-5 kHz (Fig. 8A). Such frequencies are within the range of what can be achieved by simple

abdominal tremulation [3, 16-19, 58], in the absence of complex elastic recoil devices. The call of *P. hammoniorum* can be subdivided into two distinct stages – a regular song and a climax song (Fig. 8B). The main component of the regular song is a pulse of high fundamental frequency (500 Hz-4 kHz) that sometimes is preceded by a sequence of several pulses of low fundamental frequency (500-1000 Hz; Fig. 8C, D). The pulses with the high frequency follow each other ca. every second. The climax song, which typically lasts only for about 10 seconds (Fig. 8B), is essentially a sped-up version of the regular song (pulse frequency of 3-4 Hz), that lacks the intermediate low frequency pulses of the latter and consists only of high frequency pulses (Fig. 8E). Although the dominant frequency of the high frequency pulses of the climax is the same with the those of the regular song, the fundamental frequency of the former is slightly lower (3 kHz; Fig. 8F) than the latter. After the climax song, the animal either resumes the regular song, or stops singing altogether.

Figure 8. The call of the South American moss bug *Peloridium hammoniorum*, recorded using laser Doppler vibrometry. A) Spectrogram; B) wave form of courtship call, its two distinct phases – the regular song, and the climax song; C) snippet of two pulses of the regular song, with arrows emphasizing the series of low frequency pulses that precede the high frequency pulse; D) spectrogram of the same snippet; E) snippet of nine pulses of the climax song, showing that it comprises entirely of pre-pulses and high frequency pulses; F) spectrogram of the same snippet.

Analysis of video recordings of singing animals show that each vibrational cycle of the regular song begins with the abdomen in its relaxed position (Fig. 9A; Video S1). The abdomen is then raised very slightly (Fig. 9B; Video S1) and returns to its relaxed position again (Fig. 9C; Video S1). We suggest that these low amplitude motions are responsible for the low frequency pulses of the regular song (Fig. 8B, C). At some point the abdomen is lifted considerably higher than before (Fig. 9D), whose movement corresponds to the distinct pre-pulse prior to each high

frequency pulse (Fig. 9D). The high frequency pulse is generated once the abdomen rapidly returns to its original relaxed position (Fig. 9E), and the song cycle is completed.

Figure 9. Stages of abdominal tremulation of a male *Peloridium hammoniorum* (left) and their corresponding stages in the waveform of the courtship song (right): (A) relaxed, (B) low frequency pulse, (C) relaxed, (D) pre-pulse; (E) high frequency pulse (end of cycle). Green and blue circles indicate position of other selected areas of the snapping organ in the current and previous panel, respectively. The red box on the waveform panel indicates vibrational activity associated with the stage of motion represented in that panel. The displacement axis is the same in all panels.

Although we do not have direct recordings of muscle activity, the only muscles that are anatomically capable of lifting the abdomen upwards are certain DVMs (IIIdvm8) and DLMs (dlmI), while the downwards motion is caused by DVM relaxation and contraction of VLMs, (IIIvlm2, vlmII), which are the primary retractors of the abdomen [59-61]. This function is further supported by the presence of apodemes 1-2 on the abdominal sterna, which serve as attachment sites for the VLMs (IIIvlm2, vlmII) (Fig. 7A), suggesting that these regions undergo considerable mechanical stress. The differences in amplitude between the low frequency and high frequency pulses may be accomplished by different degrees of contraction of the three muscles involved, or differential contraction in the levators (only IIIdvm8 or dlmI contracts in low frequency pulse, while both contract concurrently during the high frequency pulse). If our interpretation of the mechanism is accurate, then the climax song is achieved solely by the high amplitude abdominal motions that we propose are responsible for the high frequency peaks of the regular song (Fig. 9D). Directing the laser on the moss bugs' body in an attempt to measure possible contractions of muscles or changing geometry of sclerites caused the animals to stop singing and triggered an escape response, which prevented us from obtaining data on the energetics of vibration production in these insects.

Overall, neither the internal and external morphology of moss bugs nor their vibrational song suggest the presence of an elastic recoil device, indicating that simple abdominal tremulation is the underlying mechanism. Hence, referring to the mechanism of Coleorrhyncha as a tymbal (a ribbed, buckling structure, primarily operated by DVMs) [23, 45] is morphologically and functionally imprecise. The functional role of the posterolateral ridge (PLR) of tergite 1 remains poorly understood. The function of a spring that brings the abdomen back into position is unlikely since the PLR is not sclerotised, nor do muscles attach directly onto it (Fig. 7B), and it does not display blue fluorescence under CLSM (Fig. 3B, D), as high-stress cuticle with resilin does [20-22]. We suggest that a more likely function of the PLR is that of a point of weakness that enables successful deformation of tergite one upon contraction of muscle dlmI.

Deep time morphological conservatism of the moss bug vibrational mechanism

We next sought to examine the pregenital abdominal morphology in extinct relatives of moss bugs. We focused on two exceptionally preserved fossils, *Karabasia evansi* Popov & Scherbakov, 1991 (Karabasiidae) and *Hoploridium dollingi* Popov & Scherbakov, 1991 (Hoploridiidae), dated to the Late Jurassic (circa 160-145 mya) and the Early Cretaceous (145-100 mya), respectively. Recent phylogenies suggest that Hoploridiidae is the sister group to Peloridiidae, whereas Karabasiidae was recovered as sister to the Hoploridiidae-Peloridiidae clade [33], even though these proposed affinities are in need of further study [34]. Our examination revealed remarkable morphological conservatism in the abdomen of Coleorrhyncha.

In *K. evansi* the dorsal surface of the abdomen (Fig. 10A, B) is nearly identical to that of *Peloridium* (Fig. 10C), including the long but rounded tergite one, a key element of the vibrational mechanism (as it contains the DLMs, the principal levators of the abdomen), and

the strip-like tergite two. As for *H. dollingi*, its dorsal abdominal morphology is not preserved, but the ventral one (Fig. 10D, E) does not differ from that of extant Coleorrhyncha (here exemplified by *Hemiodoecus leai*, Fig. 10F), particularly in the morphology of the laterotergites and the position of the spiracles. We suggest that *H. dollingi*'s dorsal structure of abdomen is unlikely to differ from that of extant Coleorrhyncha. Details of the internal musculature are also not preserved in these fossils, nor is it possible to reconstruct the biomechanics of vibration production. However, based on the data presented here, it is evident that the gross abdominal structure of Coleorrhyncha, including those parts involved in vibrational signal production (tergites one and two) in modern forms, has remained largely unchanged for more than 145 million years. It remains to be found whether presumed stem-Coleorrhyncha, such as the family Progonocimicidae, possessed the same vibrational mechanism. We were unable to find fossil Progonocimicidae where the basal portion of the pregenital abdomen is visible, while their affinities to peloridiids are considered doubtful by some authors [34].

Figure 10. Comparison of the pregenital abdominal morphology of extinct moss bug relatives to that of extant species. A) Ventral surface of *Karabasia evansi* (holotype male, 3015/380), with dorsal structures (tergites 1-2) visible, imaged with scanning electron miscroscopy; B) same, with an illustrated interpretation of its morphology; C) scanning electron microscopy image of the dorsal pregenital abdomen of the extant species *Hemiodoecus leai*; D) ventral surface of *Hoploridium dollingi* (holotype female 1989/3555), imaged with scanning electron miscroscopy; E) same, with a schematic reconstruction of its morphology and how it relates to; F) the ventral abdominal morphology of the extant species *H. leai*. Note that the holotype of *H. dollingi* is a female, whereas the extant specimen of *H. leai* is male. The pregenital abdominal morphology is largely identical in both sexes. Abbreviations : crn = median carina ; ltg = laterotergite ; sp = spiracle ; t = tergite. (Photographs of the fossils courtesy Roman Rakitov and Dmitry Shcherbakov).

Phylogenetic analysis

We next sought to incorporate the newly described morphological characters of the peloridiid vibrational mechanism in a morphological matrix used previously for a phylogenetic analysis [62]. Two phylogenetic hypotheses were produced – one with just 18 characters of the pregenital abdomen (Fig. 11A), and one with the latter integrated with 93 previously used SEM characters described in [62] (Fig. 11B).

Figure 11. Results of maximum parsimony phylogenetic analyses performed in TNT on different sets of characters. Numbers above the nodes represent bootstrap values, below – the Bremer support values. A: 18 characters of the pregenital abdomen pertaining to the putative vibroacoustic apparatus; B: characters from matrix A integrated into the matrix of 93 morphological characters used in (63), involving only the taxa used in both (63) and this study.

The traditional search of the most parsimonious trees on a matrix including only the characters of the pregenital abdomen (Supplementary file 3A) resulted in a single most parsimonious tree (9.605 rearrangements, best score 24, best score hit 10 times out of 10). In the resulting tree, Auchenorrnyncha and Heteroptera were retained as monophyletic, although Heteroptera with only 27 in bootstrap and 1 in Bremer support value (Fig. 11A). The four peloridiid species remained in the polytomy, but formed a monophyletic group together with Heteroptera – although here as well, with only 34 bootstrap and 1 Bremer support value (Fig. 11A).

The integrated set (Fig. 11B) with the characters of the pregenital abdomen and the previously used SEM characters (Supplementary file 3C), when analysed in traditional search with the same parameters, resulted also in a single most parsimonious tree (7.623 rearrangements, best score 167, hit 10 times out of 10) (Fig. 11B). In this tree, Heteroptera, Auchenorrhyncha and Peloridiidae are all recovered as monophyletic (Fig. 11B), with Peloridiidae having the least support (69 bootstrap, 2 Bremer support). As in the analysis by [62] (Supplementary file 3B), performed exclusively with SEM characters, Peloridiidae was recovered as the sister group of Auchenorrhyncha, with a bootstrap value of 75 and Bremer support of 5 (Fig. 11B).

Discussion

In the present work, we attempted to elucidate the evolution of complex vibroacoustic mechanisms in Hemiptera. To this end, we revealed the previously unknown functional morphology of the evolutionarily important and biomechanically unstudied moss bugs, and we studied the bearing of this new morphological information for their phylogenetic relationships to other Hemiptera. We find that the inclusion of vibrational organs in phylogenetic matrices may be a useful approach for testing existing hypotheses on hemipteran systematics. Some new phylogenomic studies indicated that Peloridiidae are the sister group to Auchenorrhyncha [52, 54]. The characters of the pregenital abdomen and putative vibrational organ, when considered alone, support a closer relatedness to the true bugs (Fig. 11A), in accordance with the more traditional phylogeny of Hemiptera [51], although the statistical support was quite poor in this case (Fig. 11A). However, when the morphological matrix comprising abdominal characters is merged with a previously published matrix [62], Peloridiidae are recovered as forming a monophyletic group with Auchenorrhyncha (Fig. 11B), in agreement with the latest phylogenomic studies [52, 54].

Regardless of topology, the most parsimonious hypothesis is that the moss bugs ancestrally lack elastic recoil devices, the latter evolving only in Auchenorrhyncha (whether the snapping organ of planthoppers and the tymbals of cicadas represent homologous or independently evolved mechanisms remains to be proven [23]). Although we cannot exclude that Peloridiidae ancestrally possessed elastic recoil devices but lost them secondarily, this is highly unlikely. The abdomen of putative extinct relatives of peloridiids from the Late Jurassic-Early Cretaceous displays remarkable morphological similarity to the vibrational organ of modern moss bugs, suggesting that this is the ancestral vibrational mechanism for the principal groups of Coleorrhyncha. Furthermore, loss of elastic recoil devices in Hemiptera is remarkably rare, represented by only five instances of tymbal-less cicadas [65-67]. Even in this case, however,

at least two of the tymbal-less cicadas compensate for this loss by producing acoustic sound through percussion [67], thereby showing the behavioural significance of vibroacoustic organs and elastic recoil devices in the Auchenorrhyncha.

Based on the above, we suggest that simple abdominal tremulation likely first appeared in the common ancestor of Euhemiptera, which is dated to the Carboniferous (350 mya [52]), in accordance with previous studies [6, 16, 55, 63, 64]. Tremulation may therefore represent one of the oldest and most widely distributed modes of vibrational signalling in the animal kingdom.

If the above hypothesis is correct, the vexing question is to determine the selective pressures that led to the evolution of elastic recoil devices in Auchenorrhyncha. Elastic recoil mechanisms allow microscopic animals to overcome the limitations of their size and enable long-range transmission of sensorially efficient vibrational signals [68, 69]. However, generating vibroacoustic signals with elastic recoil devices may be energetically costly [70], while the sheer anatomical complexity of tymbals and snapping organs [21, 22] may require a series of morphological transformations that are developmentally challenging to achieve. Although hemipteran abdominal tremulation may represent a biomechanically efficient means of generating vibrational signals for more than 350 million years, the emitted calls are rather uniform (Fig. 8; [4, 6]). Insects singing in sexual contexts are under strong selective pressure to enhance their vibroacoustic repertoire with more complex songs [71], yet abdominal tremulation might be limited in the number of distinct signals that it can produce [16]. To overcome this constraint, Hemiptera supplement their tremulatory courtship songs with percussion [17, 18], wing buzzing [19], and an extremely diverse set of stridulatory mechanisms [16]. Likewise, we propose that elastic recoil devices such as the snapping organ and the tymbal may have arisen in Auchenorrhyncha due to intense sexual selection for more

complex courtship signals, which would have been impossible to achieve by simple abdominal tremulation alone.

In conclusion, our study addresses one of the last outstanding knowledge gaps in hemipteran vibroacoustic signalling evolution. At the same time, we provide novel morphological and generalized data that offer themselves for further hypothesis testing on the behavioural, physical, and developmental drivers that led to the diversity of hemipteran vibroacoustic mechanisms that we observe today. Understanding the developmental pathways that lead to the development of hemipteran elastic recoil devices will be essential towards resolving their homologies.

Acknowledgements

This publication arises from research funded by the Leverhulme Trust Early Career Fellowship grant (ECF-2021-199) and the John Fell Oxford University Press Research Fund to L.-R. Davranoglou. V. Hartung was funded by the Elsa Neuman doctorate grant (application number H49023) and the German Academic Exchange travel grant (processing number D/09/04219). The authors thank Kristin Mahlow (Berlin Museum of Natural History) for obtaining micro-CT scans of *P. hammoniorum* and *P. pomponorum*. The authors acknowledge the Paul Scherrer Institut, Villigen, Switzerland for provision of synchrotron radiation beamtime at beamline TOMCAT X02DA of the SLS. The authors are grateful to Dávid Rédei for his helpful comments on peloridiid morphology, and to Dimitri Scherbakov and Roman Rakitov for providing the scanning electron images of the fossil specimens and informative discussions of the fossils' morphology. Jürgen Deckert kindly provided video and photo materials of Peloridiidae. André Nel provided rare papers on fossil Coleorrhyncha. Nature protection officials in Australia, Chile and New Zealand provided collection and export permits (Material and Methods).

S1 Table. A list of all the moss bug species examined and their label information.

Species	Specimen collection site	Analysis specimen was used in
Hackeriella brachycephala	Australia, New South Wales, New England National Park, 03-07.12.2009, sifted from *Dicranoloma dicarpum*, coll. Viktor Hartung	SEM
Hackeriella veitchi	Australia, Springbrook National Park, summer 2007, from bryophytes, coll. Geoff Monteith	SEM
Hemiodoecus leai	Australia, New South Wales, Kosciuszko National Park, Rennix Gap, S 36° 21,599’, E 148° 30,482’; elevation 1566 m. 18-27.12.2009, from *Sphagnum cristatum*, coll. Viktor Hartung	SEM, synchrotron micro-CT, photomicrography, confocal laser scanning microscopy
Oiophysa cumberi	New Zealand, North Island, Tongariro National Park, 16.04.2010, from *Ptychomnion aciculare*, *Leucobryum candidum*, *Echinodium hispidum* and *Camptochaete arbuscula*, coll. Viktor Hartung	SEM
Peloridium hammoniorum	Chile, Chiloe, Estacion Biologica „Senda Darwin“, Ancud, 02.02.2014, from *Polytrichadelphus magellanus*, coll. Viktor Hartung	SEM, benchtop micro-CT, laser Doppler vibrometry, videography
Xenophyes cascus	New Zealand, North Island, Orongorongo, Rimutaka forest park, 06.04.2010, from *Dicranoloma dicarpum* , *Dicranoloma billarderi*, *Plagiochila stephensoniana* and *Ptychomnion aciculare*, coll. Viktor Hartung; Otaki forks, Tararua forest park, 09-12.04.2010, from unspecified bryophytes and *Racopilum convolutaceum*, colls. Viktor Hartung & George Gibbs; Taranaki National Park, Dawson Falls, 14.04.2010, from *Leucobryum candidum* and *Dicranoloma* sp., coll. Viktor Hartung; Tongariro National Park, 16-17.04.2010, from *Ptychomnion aciculare*, *Leucobryum candidum*, *Echinodium hispidum* and	SEM, synchrotron micro-CT, confocal laser scanning microscopy

	Dendrohypopterygium filiculiforme, coll. Viktor Hartung	
Xenophyes kinlochensis	New Zealand, South Island, Fiordland National Park, Key Summit track, 06-08.03.2010. from *Chandonanthus squarrosus*, *Plagiochila circinalis*, *Ptychomnion aciculare*, and *Sphagnum cristatum*, coll. Viktor Hartung	SEM
Xenophysella stewartensis	New Zealand, Stewart Island, Horseshoe Bay, 13-17.02.2010, from *Bazzania adnexa*, *Dicranoloma billardieri*, and *Schistochila lehmanniana*, coll. Viktor Hartung	SEM

S2 Table. List of the muscles associated with the moss bug vibrational organ and their attachments.

S1 video. Videography of a male *Peloridium hammoniorum* generating its call.

S3 Supplementary files. Data matrices of the characters used in the phylogenetic analysis. A) 18 characters of the pregenital abdomen, obtained in this study, used to produce the phylogenetic hypothesis in Fig. 11 A. B) 93 characters of microscopic characters obtained with SEM (63). C) An integrated set of the matrices A and B, with only those taxa retained whose character states were known for both matrices, used to produce the phylogenetic hypothesis in Fig. 11 B.

Material and Methods

Field work. Peloridiidae specimens were collected in Australia in 2009-10, in New Zealand 2010 and in Chile 2014 – bryophyte samples were first sifted and then the sifted material was analyzed for up to 24 hours (depending on the moisture) in Berlese funnels. Collections in

Australia were done under permits № WITK06355209 (Queensland), S13005 (New South Wales), 10005138 (Victoria) and FA10018 (Tasmania); in New Zealand under WE-26346-RES; in Chile under a permit issued by CONAF.

Bioacoustic recordings. Acoustic signals of Peloridiidae were recorded with the Polytec PDV100 vibrometer and a Roland digital recorder (sampling rate: 44100 Hz, bit depth: 16 bit). Recordings were made in closed plastic vials with wet bryophytes with the peloridiid specimens sitting on them. The laser beam of the vibrometer was directed at a small piece of reflector foil glued to the stem of the bryophyte where the specimens were sitting. Oscillograms were analyzed with Audacity 2.1.3 and spectrograms were constructed using Raven Lite 2.0 (Cornell Lab of Ornithology).

Confocal Laser Scanning Microscopy (CLSM). Specimens of *Hemiodoecus leai* and *Xenophyes cascus* were placed between two cover slips in 70% ethanol. Images were taken with an Olympus FV1000, at a laser wavelength of 488 nm.

Scanning electron microscopy (SEM). All specimens of Peloridiidae were first manually cleaned using a paintbrush and fine needle after overnight incubation in ethyl acetate. The specimens were then critically-point dried on a BAL-TEC CPD 030, sputtercoated on Quorum SC7640 and Quorum Q150RS. Scanning electron microscopy was performed on a Zeiss EVO LS10. Fossil Coleorrhyncha were imaged by Roman Rakitov with a Tescan Vega3 scanning electron microscope at the Paleontological Institute, Russian Academy of Sciences.

Microcomputed tomography (micro-CT). Three *H. leai* and one *X. cascus* ethanol-preserved specimens were scanned at the TOMCAT beamline, Swiss Light Source (SLS), Paul Scherrer Institut, Switzerland, at a beam energy of 16 keV with final pixel size of 0.65 um. Additionally, two ethanol-preserved specimens of *P. hammoniorum* and *P. pomponorum* were

subjected to micro-tomographic analysis at the Museum für Naturkunde Berlin (SCR_022585) using a Phoenix nanotom X-ray tube (Waygate Technologies, Baker Hughes, Wunstorf, Germany; SCR_022582) at 70kV and 150 or 200µA, generating 1000 projections with 750ms per scan. The effective voxel size was 4,2 um. The cone beam reconstruction was performed using the datos|x 2 reconstruction software (Waygate Technologies, Baker Hughes, Wunstorf, Germany; datos|x 2.2) and the data were visualized in VG Studio Max 3.5 (Volume Graphics GmbH, Heidelberg Germany).

Three-dimensional reconstruction of both the synchrotron and benchtop micro-CT scans was undertaken using Amira 6.1 software (Mercury Systems).

Photomicrography. A male ethanol-preserved specimen of *H. leai* was imaged with a Leica M165c microscope equipped with a Leica DFC490 camera. The resulting stacked images were combined using Helicon Focus (Helicon Soft, Kharkiv, Ukraine) or VHX-5000 system software.

Videography. Records of two Chilean specimens of *P. hammoniorum* were performed on native *Polytrichadelphus* moss at room temperature, using a Canon 5D Mark II camera (30 frames per second), MP-E 65mm lens and Macro Ring Lite MR-14EX cold light source.

Phylogenetic analysis. 18 characters of the pregenital abdomen that pertain to the body regions normally involved in acoustic signalling in Hemiptera, were coded in a phylogenetic matrix for four peloridiid species and several representatives of other three hemipteran suborders (Supplementary file 3A). Character matrices for phylogenetic analysis were produced with WinClada 1.00.08 [72]. The matrix of 93 morphological characters established with scanning electron microscopy [62] was used in the analysis along with 18 morphological characters

based on the study of pregenital abdomen described in this paper. The 18 abdominal characters were analysed separately and also as an integrated data set with the 93 SEM characters.

Phylogenetic analysis was performed with TNT version 1.1 (sponsored by the Willi Hennig Society, [73]), one of the most efficient packages utilizing maximum parsimony methods. *Psylla alni* was set as the root; characters were unweighted and non-additive. Traditional search was performed with default settings (Wagner trees, 1 random tree, 10 replications, TBR (tree bisection reconnection) swapping algorithm, 10 trees to save per replication). Every time a new traditional search was performed, the program was started anew to avoid influence of trees that could be stored in the buffer.

Bremer support values were counted by TBR from existing trees, retaining trees suboptimal by 20 steps. Images provided by TNT were enhanced using Adobe® Illustrator® 2021 and Adobe® Photoshop® 2021.

References

1. Cocroft RB, Rodriguez, RL. The behavioural ecology of insect vibrational communication. Bioscience. 2005; 55: 323-334.

2. Čokl A, Virant-Doberlet M. Communication with substrate-borne signals in small plant-dwelling insects. Annu Rev Entomol. 2003; 48: 29-50.

3. Virant-Doberlet M, Čokl A. Vibrational communication in insects. Neotrop Entomol. 2004; 33: 121-134.

4. Moraes MCB, Laumann RA, Čokl A, Borges M. Vibratory signals of four Neotropical stink bug species. Physiol Entomol. 2005; 30: 175-188.

5. Gemeno C, Baldo G, Nieri R, Valls J, Alomar O, Mazzoni V. Substrate-borne vibrational

signals in mating communication of Macrolophus Bugs. J Insect Behav. 2015; 28: 482-498.

6. Gogala M. Vibratory signals produced by Heteroptera - Pentatomorpha and Cimicomorpha. In: Drosopoulos S, Claridge M, editors. Insect sounds and communication: physiology, behavior, ecology, and evolution (Contemporary Topics in Entomology). CRC Press; 2006. pp. 275-295.

7. Legendre F, Marting PR, Cocroft R. Competitive masking of vibrational signals during mate searching in a treehopper. Anim Behav. 2012; 83: 361-368.

8. Elias DO, Kasumovic MM, Punzalan D, Andrade MCB, Mason AC. Assessment during aggressive contests between male jumping spiders. Anim Behav. 2008; 76: 901-910.

9. Delattre O, Šobotník J, Jandák,V, Synek J, Cvačka J, Jiříček O, et al. Chemical and vibratory signals used in alarm communication in the termite Reticulitermes flavipes (Rhinotermitidae). Insect Soc. 2019; 66: 265-272.

10. Cocroft RB. Parent-offspring communication in response to predators in a subsocial treehopper (Hemiptera: Membracidae: Umbonia crassicornis). Ethology. 1999; 105: 553-568.

11. Cocroft RB. Vibrational communication and the ecology of group-living, herbivorous insects. Amer Zool. 2001; 41: 1215-1221.

12. Mukai H, Hironaka M, Tojo S, Nomakuchi S. Maternal vibration: An important cue for embryo hatching in a subsocial shield bug. PLoS One. 2014; 9: e87932.

13. Henry TJ. (2017). Biodiversity of Heteroptera. In: Foottit RG. Adler PH, editors. Insect biodiversity: science and society, Vol. I, second edition. John Wiley and Sons; 2017. pp. 279-335.

14. Bartlett CR, Deitz LL, Dmitriev DA, Sanborn, Soulier-Perkins A, Wallace MS. The diversity of the True Hoppers (Hemiptera: Auchenorrhyncha). In: Foottit, RG, Adler PH.

Insect biodiversity: Science and Society, Vol. II, first edition; 2018. pp. 501-590.

15. Hardy NB. (2018). The biodiversity of Sternorrhyncha: Scale Insects, Aphids, Psyllids, and Whiteflies. In: Foottit, RG, Adler PH. Insect biodiversity: Science and Society, Vol. II, first edition; 2018. pp. 591-625.

16. Davranoglou LR, Taylor, GK, Mortimer, B. Sexual selection and predation drive the repeated evolution of stridulation in Heteroptera and other arthropods. Biol Rev. 2023; 98: 697-981.

17. Koczor S, Čokl A. Percussion signals of Lygus rugulipennis Poppius (Heteroptera: Miridae). Cent Eur J Biol. 2014; 9: 543-549.

18. Žunič A, Čokl A, Virant-Doberlet M, Millar JG, Communication with signals produced by abdominal vibration, tremulation, and percussion in Podisus maculiventris (Heteroptera: Pentatomidae). Ann Entomol Soc Am. 2008; 101: 1169-1178.

19. Kavčič A, Čokl A, Laumann RA, Blassioli-Moraes MC, Borges M. Tremulatory and abdomen vibration signals enable communication through air in the stink bug Euschistus heros. PLoS One. 2013; 8: e56503.

20. Young D, Bennet-Clark H. The role of the tymbal in cicada sound production. J Exp Biol. 1995; 198: 1001-1019.

21. Davranoglou LR, Mortimer B, Taylor GK, Malenovský I. On the morphology and evolution of cicadomorphan tymbal organs. Arthropod Struct. Dev. 2020; 55: 100918.

22. Davranoglou LR, Cicirello A, Mortimer B, Taylor GK. Planthopper bugs use a fast, cyclic elastic recoil mechanism for effective vibrational communication at small body size. PLoS Biol. 2019; 17: e3000155.

23. Wessel A, Mühlethaler R, Hartung V, Kustor V & Gogala M. The tymbal: evolution

of a complex vibration-producing organ in the Tymbalia (Hemiptera excl. Sternorrhyncha). In: Cocroft RB, Gogala M, Hill PSM, Wessel A, editors. Animal signals and communication: studying vibrational communication. Berlin: Springer; 2014. pp. 395-444.

24. Davranoglou LR, Cicirello A, Mortimer B, Taylor GK. Response to “On the evolution of the tymbalian tymbal organ: Comment on ‘Planthopper bugs use a fast, cyclic elastic recoil mechanism for effective vibrational communication at small body size’ by Davranoglou et al. 2019.” Cicadina. 2019; 18: 17-26.

25. Burckhardt D. Taxonomy and phylogeny of the Gondwanan moss bugs or Peloridiidae (Hemiptera, Coleorrhyncha). Dtsch Entomol Z. 2009; 56: 173-235. doi:10.1002/mmnd.200900019.

26. Burckhardt D, Bochud E, Damgaard J, Gibbs GW, Hartung V, Larivière M-C, Wyniger D, Zürcher I. A review of the moss bug genus Xenophyes (Hemiptera: Coleorrhyncha: Peloridiidae) from New Zealand: systematics and biogeography. Zootaxa. 2011; 2923: 1-26. doi:10.11646/zootaxa.2923.1.1.

27. Shcherbakov DE. A new species of Peloridium (Hemiptera: Coleorrhyncha, Peloridiidae) from Chile. Far East Entomol. 2014; 286: 1-11.

28. Evans JW. The biogeographical significance of the Peloridiidae (Homoptera: Coleorrhyncha) and a new species from Lord Howe Island. Proc Royal Soc Qld. 1967; 79: 17-24.

29. Evans JW. A review of present knowledge of the family Peloridiidae and new genera and new species from New Zealand and New Caledonia (Hemiptera: Insecta). Rec Aust Mus. 1981; 34: 381-406.

30. China WE. South American Peloridiidae (Hemiptera: Homoptera: Coleorrhyncha).

Ecol Entomol. 1962; 114: 131-161.

31. Szwedo J, Azar D, Ziadé K. The first Progonocimicidae (Insecta: Hemiptera: Coleorrhyncha) from Lower Cretaceous Lebanese amber. Insect Syst Evol. 2011; 42: 161-177.

32. Jiang JQ, Huang DY. New species of Cicadocoris (Hemiptera: Coleorrhyncha: Progonocimicidae) from mid-Jurassic deposits in northeastern China Eur J Entomol. 2017; 114: 355-364. doi: 10.14411/eje.2017.045 .

33. Dong QP, Yao YZ, Ren D. New fossil Progonocimicidae (Hemiptera: Coleorrhyncha: Progonocimicoidea) from the Upper Mesozoic of northeastern China, with a phylogeny of Coleorrhyncha. Syst Entomol. 2014; 39: 773-782.

34. Burckhardt D, Nel A, Raisch M & Poschmann MJ. A new putative moss bug (Insecta: Hemiptera) from the lower Permian of the Saar-Nahe Basin, SW Germany, and the age of Coleorrhyncha, Hist Biol. 2022; 35: 832-837. doi: 10.1080/08912963.2022.2067759

35. Jiang JQ, Cai CY, Huang DY. Progonocimicids from the Middle Jurassic Haifanggou Formation, western Liaoning, northeast China support stratigraphic correlation with the Daohugou beds. Alcheringa Austr J Pal, 2015; 40: 53-61. DOI: 10.1080/03115518.2015.1086053

36. Jiang T, Wang B, Szwedo J. The first representative of Progonocimicidae (Hemiptera: Coleorrhyncha) from mid-Cretaceous Burmese amber. Cret Res. 2019; 93: 346e359. https://doi.org/10.1016/j.cretres.2018.09.018

37. Martins-Neto RG, Gallego OF, Melchor RN. The Triassic insect fauna from South America (Argentina, Brazil and Chile): a checklist (except Blattoptera and Coleoptera) and descriptions of new taxa. Acta Zool Crac. 2003; 46: 229-256.

38. Szwedo J. The Coleorrhyncha (Insecta: Hemiptera) of the European Jurassic, with a description of a new genus from the Toarcian of Luxembourg. Vol Jur. 2011; 9: 3-20.

39. Wang B, Szwedo J, Zhang H C. Jurassic Progonocimicidae (Hemiptera) from China and phylogenetic evolution of Coleorrhyncha. Sci China Ser D-Earth Sci. 2009; 52: 1953-1961. doi: 10.1007/s11430-009-0160-6

40. Wang B, Szwedo J, Zhang H-C & Lin Q-B. The first Peloridioidea (Hemiptera) from China. Acta Pal Sin. 2011; 50: 321-325.

41. Kuechler SM, Gibbs G, Burckhardt D, Dettner K, Hartung V. Diversity of bacterial endosymbionts and bacteria – host co-evolution in Gondwanan relict moss bugs (Hemiptera : Coleorrhyncha : Peloridiidae). Environ Microbiol. 2013; 15: 2031-2042. https:/doi.org/10.1111/1462-2920.12101.

42. Santos-Garcia D, Latorre A, Moya A, Gibbs G, Hartung V, Dettner K, Kuechler SM, Silva FJ. Small but powerful, the primary endosymbiont of moss bugs. Genome Biol Evol. 2014; 6: 1875-1893. https:/doi.org/10.1093/gbe/evu149.

43. Hartung V, Medebach I, Walheim S. Structural plastron in relict Gondwanan moss bugs (Hemiptera : Coleorrhyncha : Peloridiidae) and its possible implications for systematics, biogeography and for the standard definition of plastron. Arthropod Struct Dev. 2016; 45: 1–10.

44. Burrows M, Hartung V, Hoch H. Jumping behaviour in a Gondwanan relict insect (Hemiptera : Coleorrhyncha : Peloridiidae). J Exp Biol. 2007; 210: 3311-3318.

45. Hoch H, Deckert, J, Wessel, A. Vibrational signalling in a Gondwanan relict insect (Hemiptera : Coleorrhyncha : Peloridiidae). Biol Lett. 2006; 2: 222-224.

46. Cryan JR, Urban JM. Higher-level phylogeny of the insect order Hemiptera: is

Auchenorrhyncha really paraphyletic? Syst Entomol. 2012; 37: 7-21.

47. Zrzavý J. Evolution of Hemiptera: an attempt at synthetic approach. Proceedings of the Sixth International Symposium on Scale Insect Studies, Part 2. 1990; pp. 19-22.

48. Song N, Liang AP. A preliminary molecular phylogeny of planthoppers (Hemiptera: Fulgoroidea) based on nuclear and mitochondrial DNA sequences. PLoS One. 2013; 8: e58400.

49. Spangenberg R, Wipfler B, Friedemann K, Pohl H, Weirauch C, Hartung V, Beutel RG. The cephalic morphology of the Gondwanan key taxon Hackeriella. Arthropod Struct Dev. 2013; 42: 1-23.

50. Friedemann K, Beutel RG. Morphology of arolia in Auchenorrhyncha (Insecta, Hemiptera). J Morphol. 2014; 275, 1217-1225.

51. Schlee D. Morphologie und Symbiose, ihre Beweiskraft für die Verwandtschaftsbeziehungen der Coleorrhyncha (Insecta, Hemiptera). Phylogenetische Studien an Hemiptera, IV: Heteropteroidea (Heteroptera & Coleorrhyncha) als monophyletische Gruppe. Stutt Beit Naturk. 1969; 210, 1-27.

52. Johnson KP, Dietrich CH, Friedrich F, Beutel RG, Wipfler B, Peters RS, et al., Phylogenomics and the evolution of hemipteroid insects. PNAS. 2018; 115, 12775-12780.

53. Yoshizawa K, Ogawa N, Dietrich CH. Wing base structure supports Coleorrhyncha + Auchenorrhyncha (Insecta: Hemiptera). J Zool Syst Evol Res. 2017; 55, 199-207.

54. Misof B, Liu S, Meusemann K, Peters RS, Donath A, Mayer C, et al. Phylogenomics resolves the timing and pattern of insect evolution. Science. 2014; 346, 763-767.

55. Davranoglou LR, Baňař P, Schlepütz CM, Mortimer B, Taylor GK. The pregenital abdomen of Enicocephalomorpha and morphological evidence for different modes of

communication at the dawn of heteropteran evolution. Arthropod Struct Dev. 2017; 46, 843-868.

56. Andersen NM. The Semiaquatic Bugs (Hemiptera: Gerromorpha). Phylogeny, Adaptations, Biogeography and Classification. Entomonograph; 1982.

57. Leston DL. The stridulatory mechanisms in terrestrial species of Hemiptera Heteroptera. Proc Zool Soc London. 1957; 128, 369-386.

58. Benediktov AA. Tremulation of firebugs Pyrrhocoris apterus L. (Heteroptera, Pyrrhocoridae). Moscow Univ Biol Sci Bull. 2007; 62, 186-187.

59. Parsons MC. Skeleton and musculature of the thorax of Gelastocoris oculatus (Fabricius) (Hemiptera-Heteroptera). Bull Museum Comp Zool. 1960; 122, 1-85.

60. Parsons MC. Thoracic skeleton and musculature of adult Saldula pallipes (F.) (Heteroptera: Saldidae). Trans R Entomol Soc London. 1963; 115, 1-37.

61. Parsons MC. Skeletomusculature of the pterothorax and first abdominal segment in micropterous Aphelocheirus aestivalis F. (Heteroptera: Naucoridae). Trans R Entomol Soc London. 1969; 121, 1-39.

62. Hartung V. Systematics of Peloridiidae (Insecta: Hemiptera: Coleorrhyncha) - an integrative approach. PhD Thesis, Humboldt Universität zu Berlin. 2018. Available from: https://doi.org/10.18452/19405

63. Gogala M. Vibration producing structures and songs of terrestrial Heteroptera as systematic characters. Biol Vestn. 1984; 32, 19-36.

64. Gogala M. Vibrational songs of land bugs and their production. In: Kalmring N, Elsner E, editors. Acoustic and vibrational communication in insects. Paul Parey Verlag. 1985; pp. 143-150.

65. Duffels JP. The systematic position of moana expansa (Homoptera: Cicadidae), with reference to sound organs and the higher classification of the superfamily cicadoidea. J Nat Hist. 1993; 27, 1223-1237.

66. Moulds MS. An appraisal of the higher classification of cicadas (Hemiptera: Cicadoidea) with special reference to the Australian fauna. Rec Aust Museum. 2005; 57, 375-446.

67. Luo C, Wei C, Nansen C. How do “mute” cicadas produce their calling songs? PLoS One; 2015; 10, 1-16.

68. Longo SJ, Cox SM, Azizi E, Ilton M, Olberding JP, St Pierre R, Patek SN. Beyond power amplification: Latch-mediated spring actuation is an emerging framework for the study of diverse elastic systems. J Exp Biol. 2019; 222, 1-10.

69. Bennet-Clark HC. Size and scale effects as constraints in insect sound communication. Philos Trans R Soc B Biol Sci. 1998; 353, 407-419.

70. Kuhelj A, de Groot M, Pajk F, Simčič T, Virant-Doberlet M. Energetic cost of vibrational signalling in a leafhopper. Behav Ecol Sociobiol. 2015; 69, 815-828.

71. Ewing AW. Acoustic signals in insect sexual behaviour. In: Lewis T, editor. 12th Symposium of the Royal Entomological Society of London, Academic Press; 1984. pp. 223-240.

72. Nixon KC. Winclada (BETA) Version 1.00.08. published by the author, Nixon KC. 1999.

73. Goloboff P, Farris J, Nixon KC. TNT, a free program for phylogenetic analysis. Cladistics. 2008; 24, 774-786.

Figure 1

Click here to access/download;Figure;Fig. 1.tif

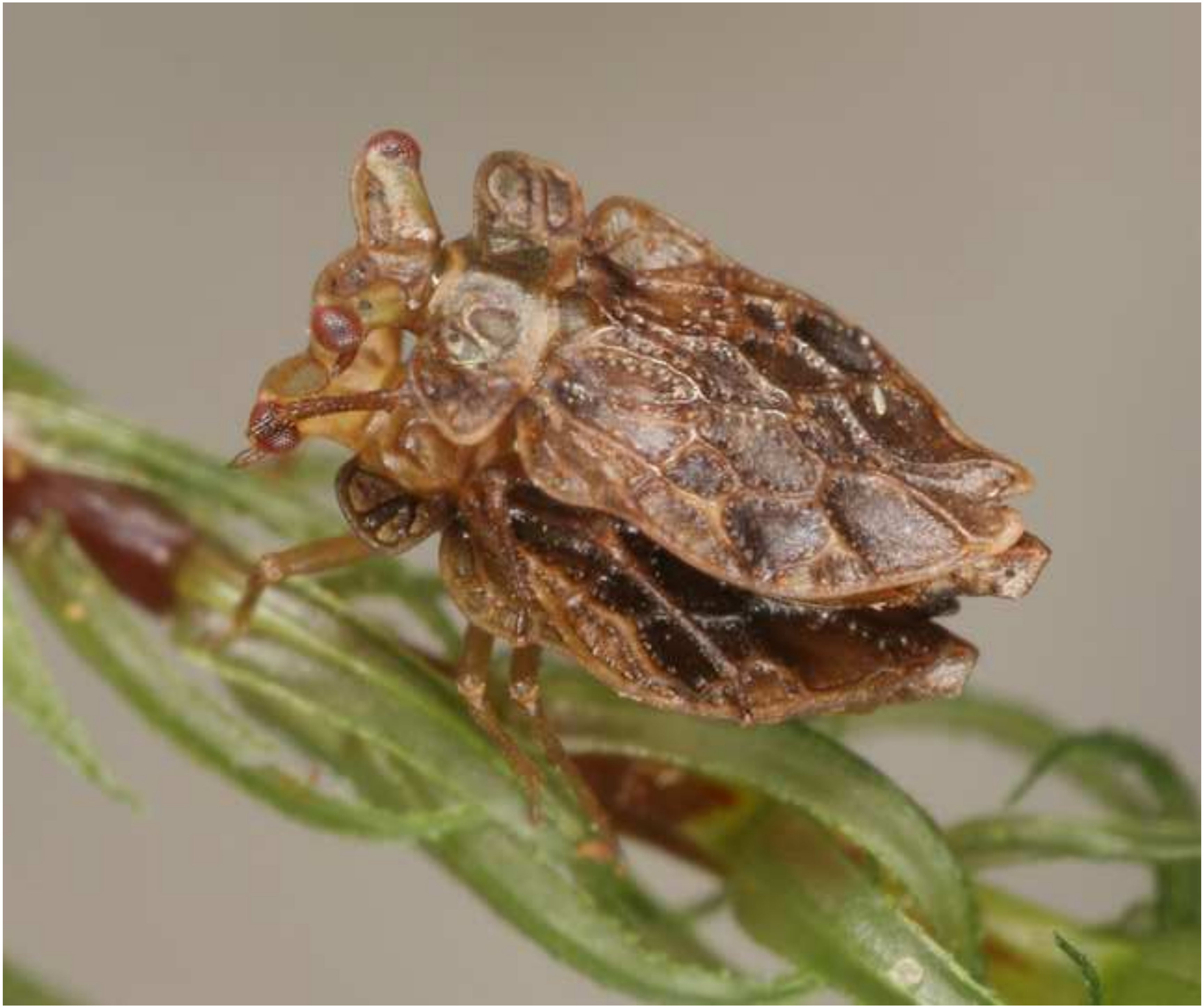

Figure 2

Click here to access/download;Figure;Fig. 2.tif

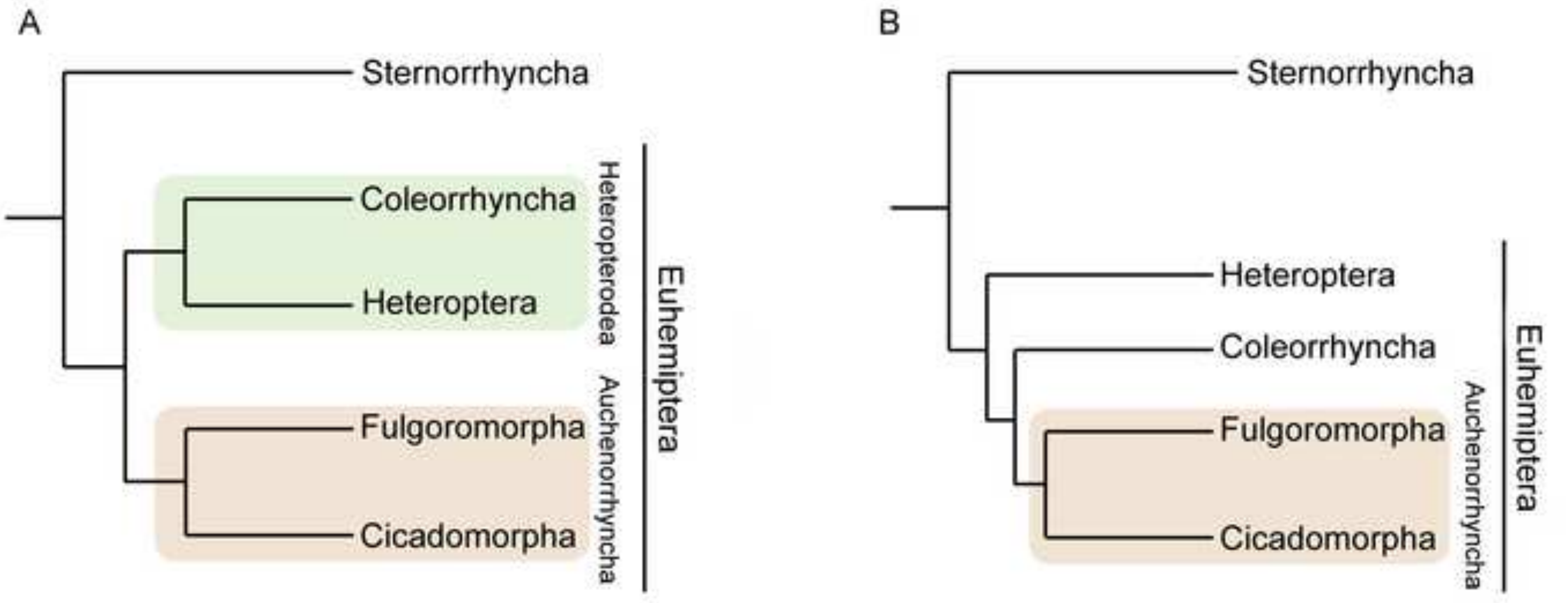

Figure 3

Click here to access/download;Figure;Fig. 3.tif

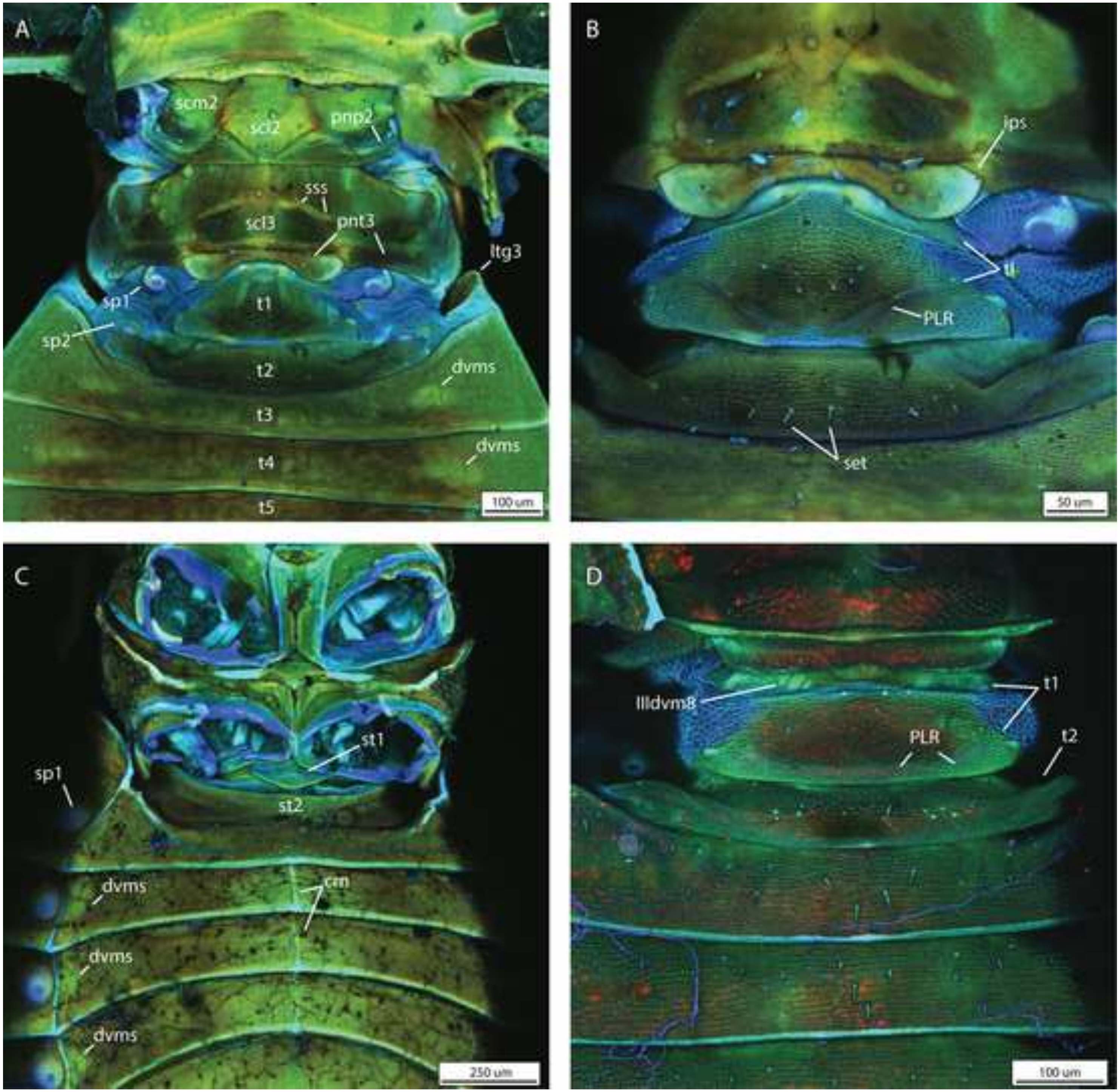

Figure 4

Click here to access/download;Figure;Fig. 4.tif

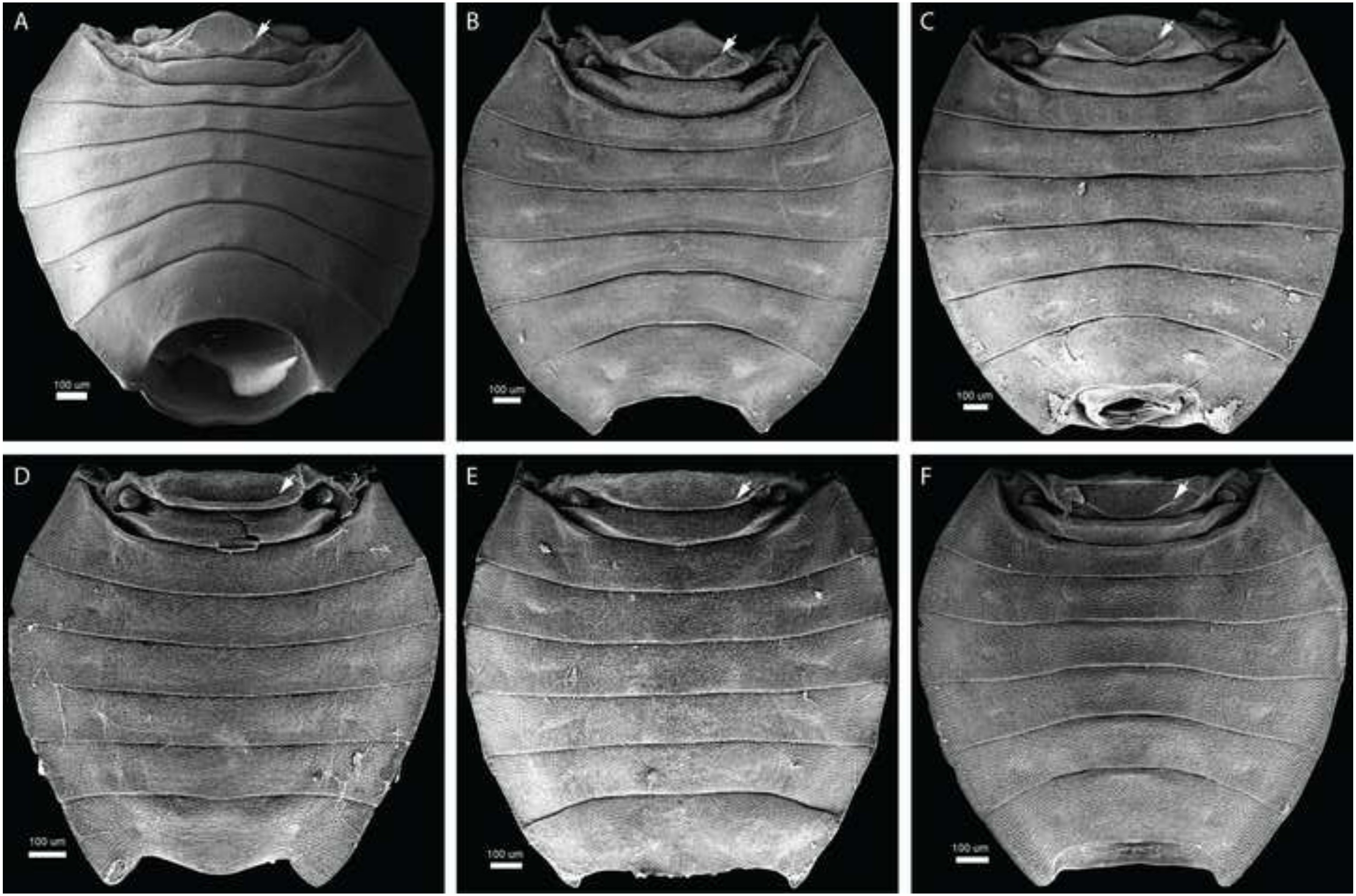

Figure 5

Click here to access/download;Figure;Fig. 5.tif

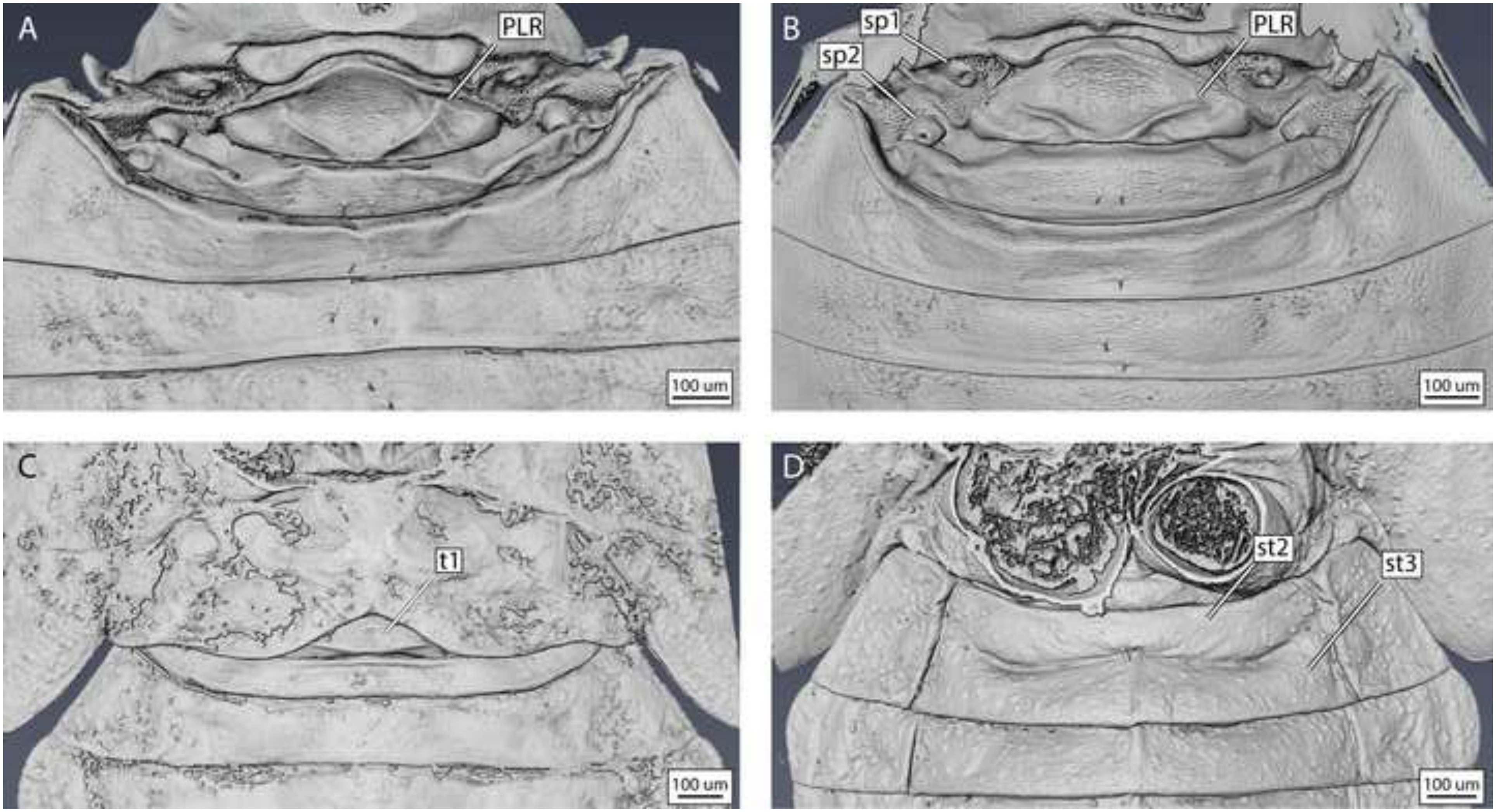

Figure 6 Click here to access/download;Figure;Fig. 6.tif

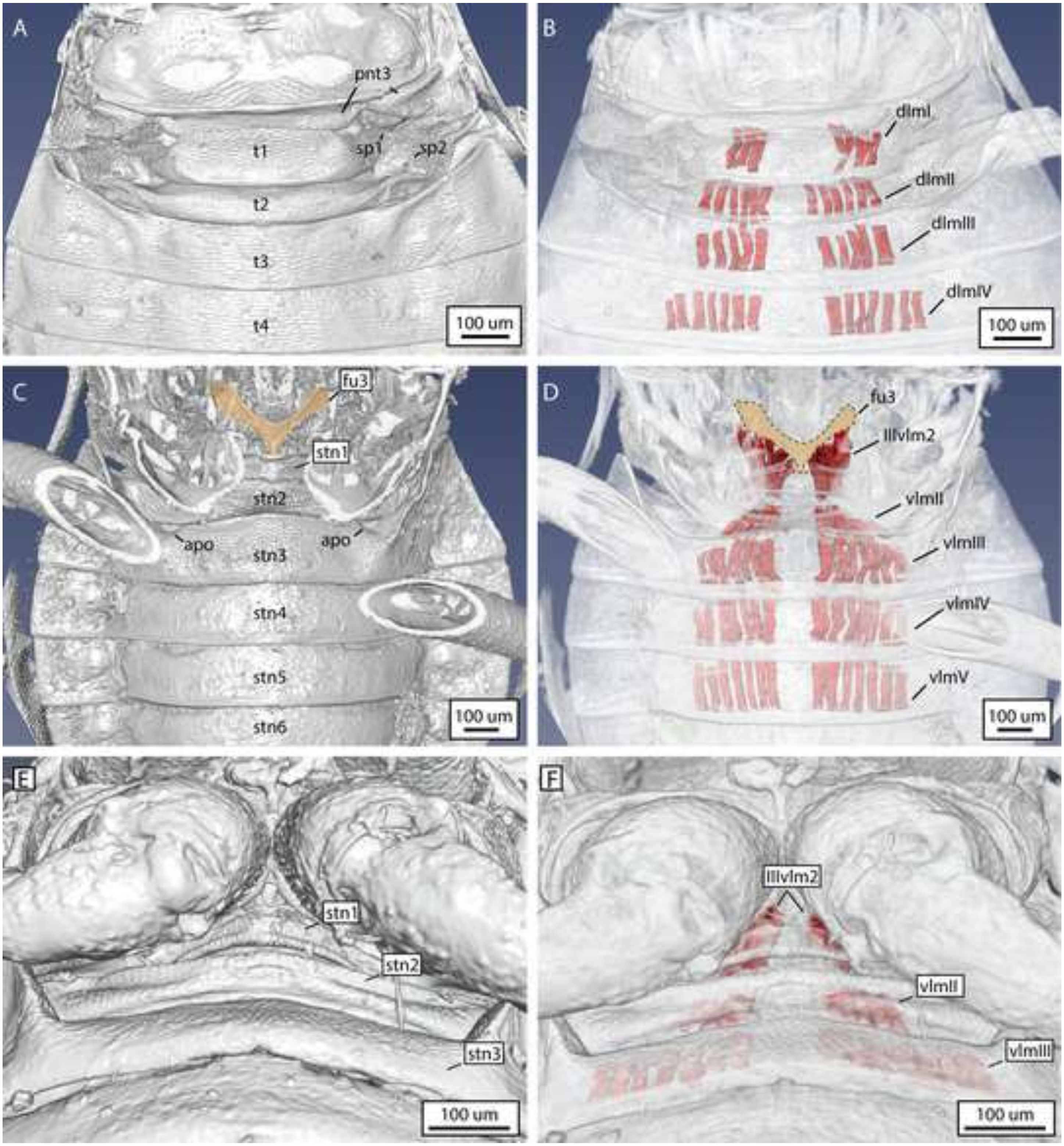

Figure 7

Click here to access/download;Figure;Fig. 7.tif

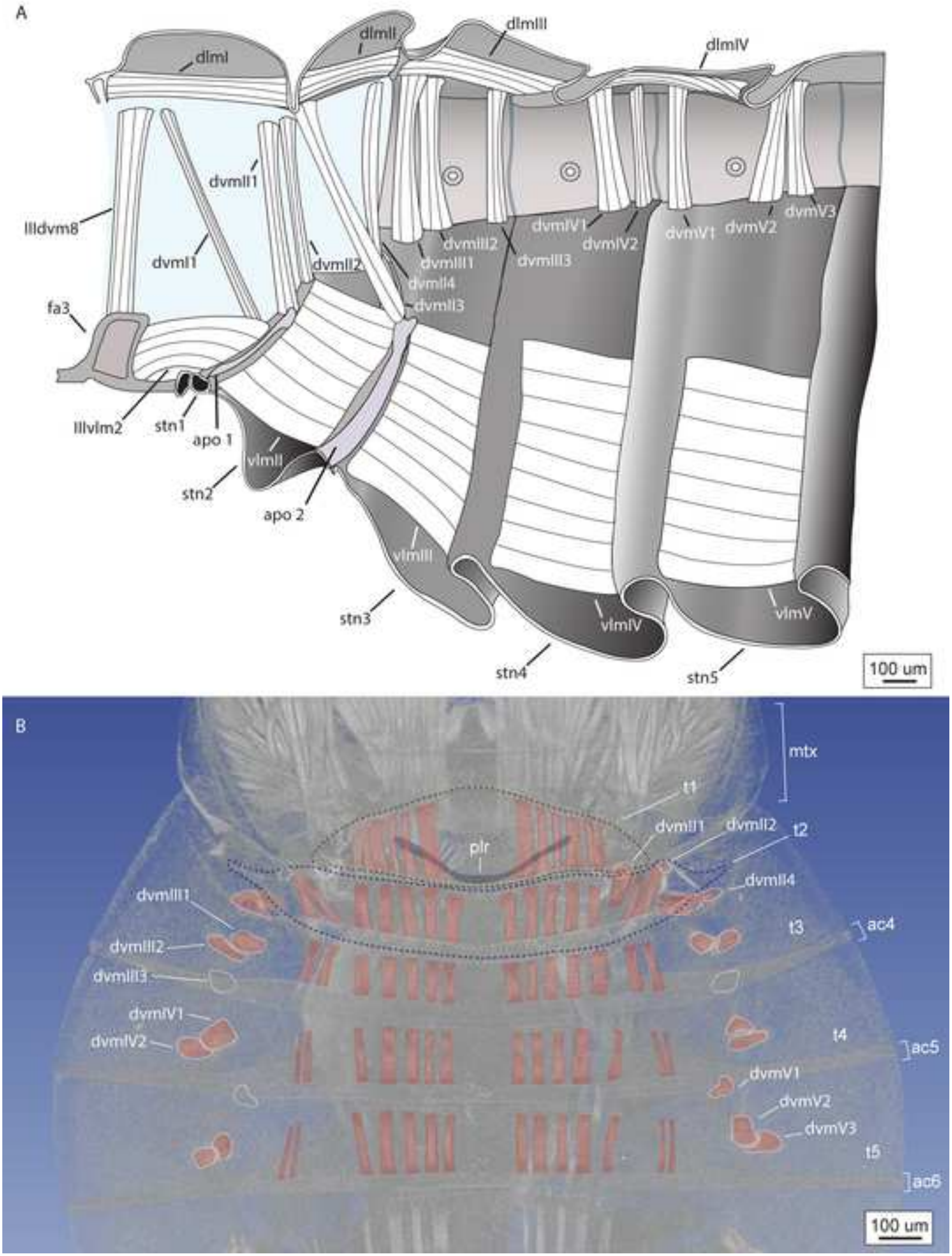

Figure 8 Click here to access/download;Figure;Fig. 8.tif

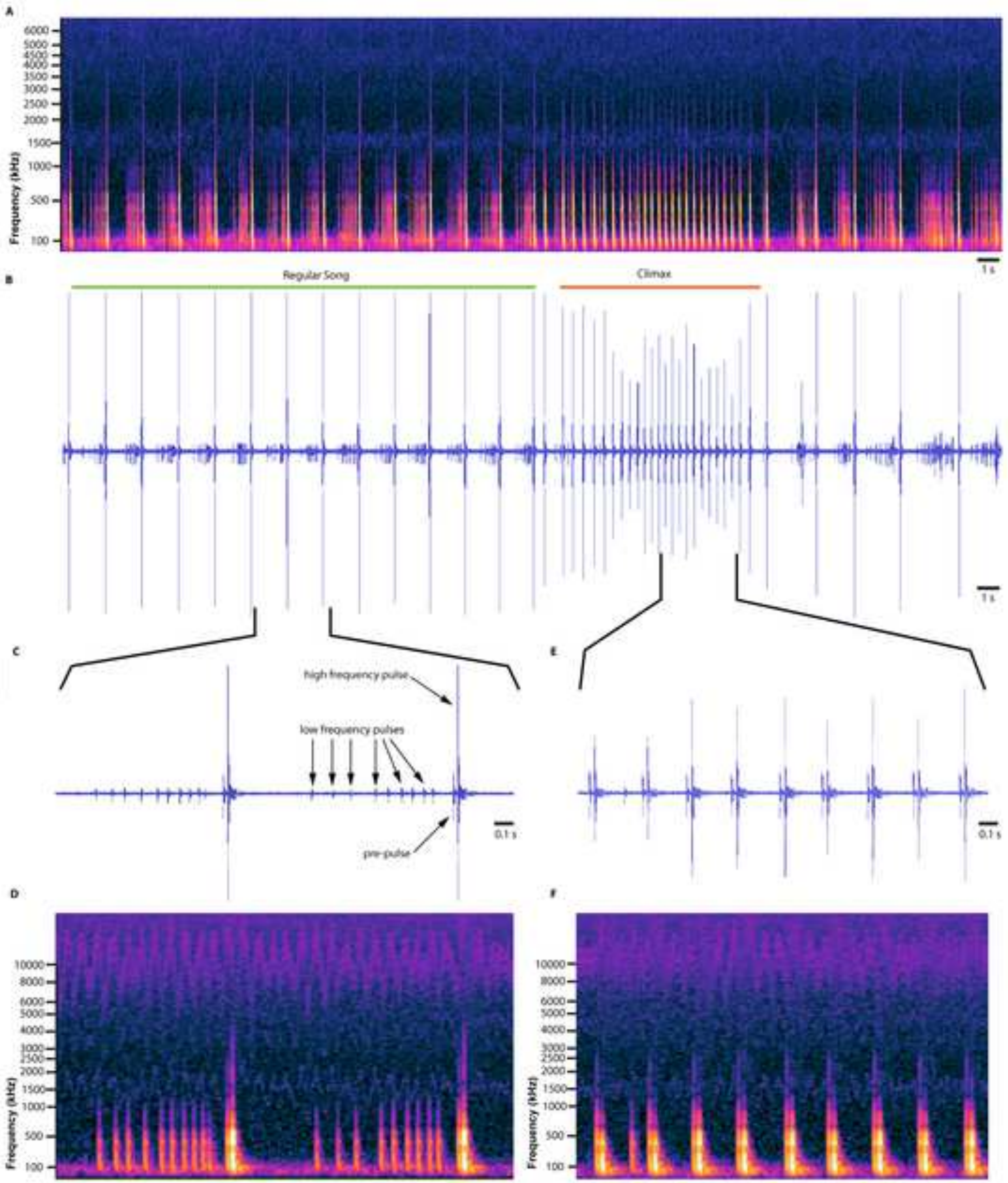

Figure 9

Click here to access/download;Figure;Fig. 9.tif

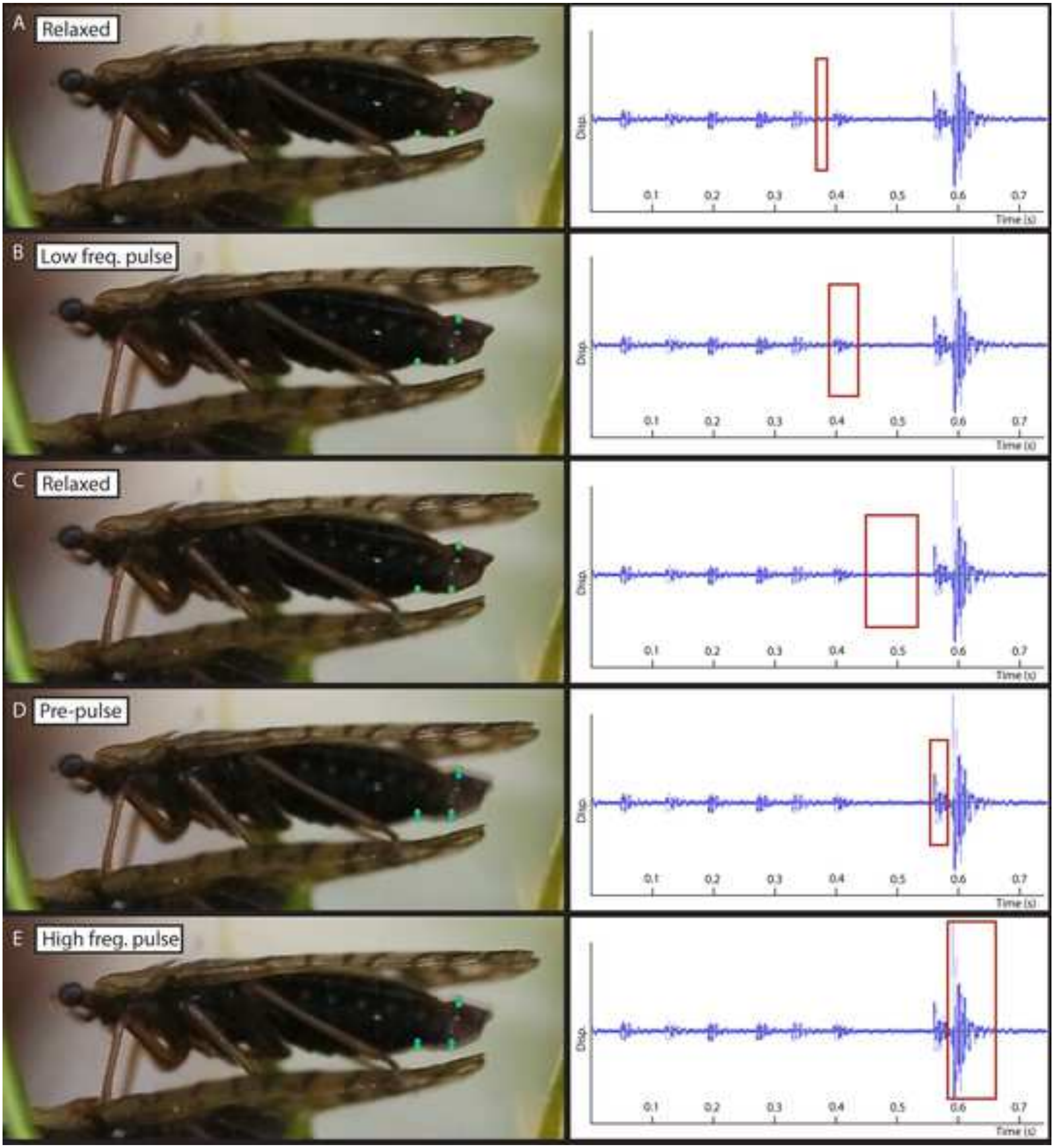

Figure 10

Click here to access/download;Figure;Fig. 10.tif

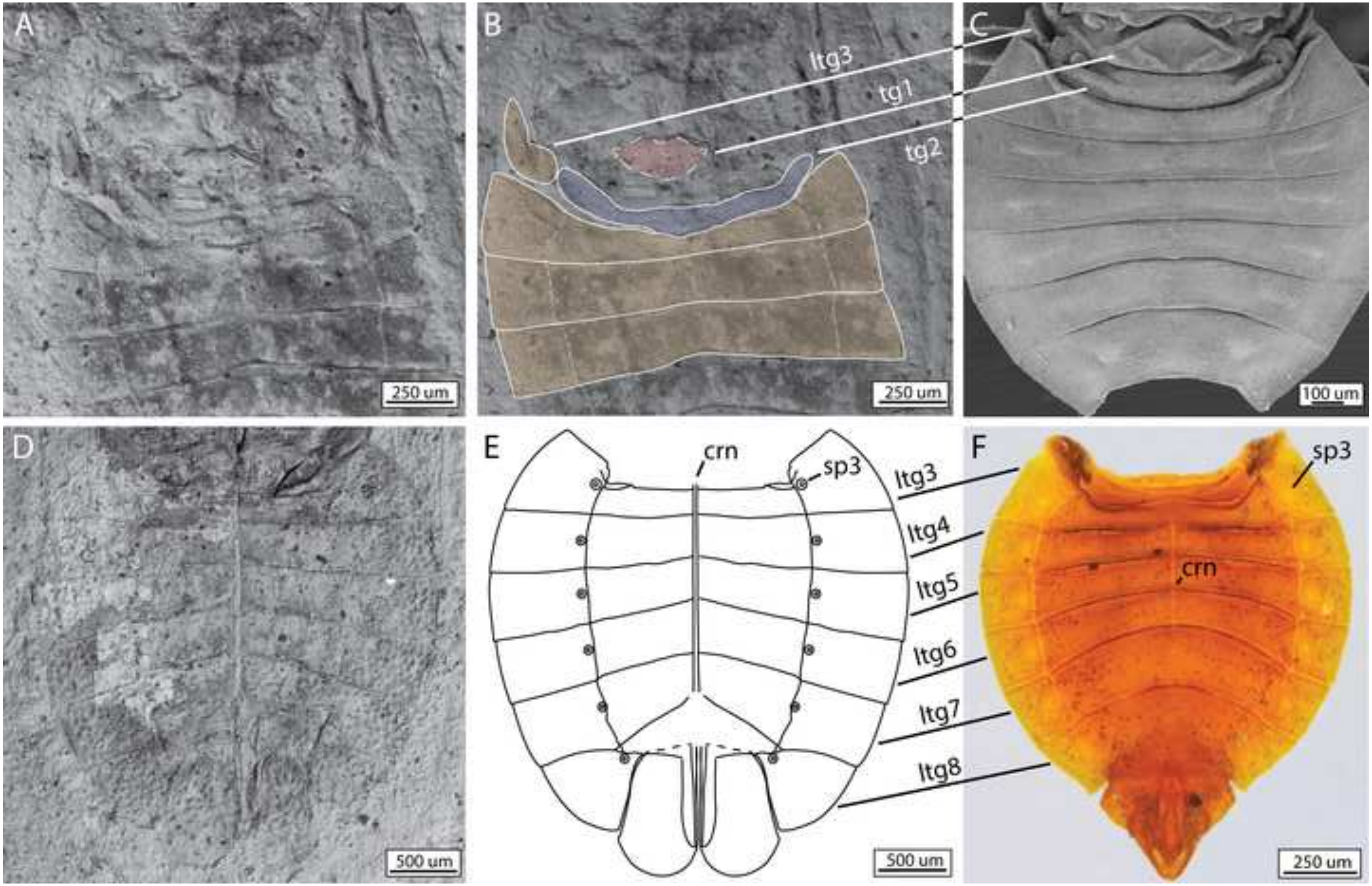

Figure 11 Click here to access/download;Figure;Fig.11-01.tif

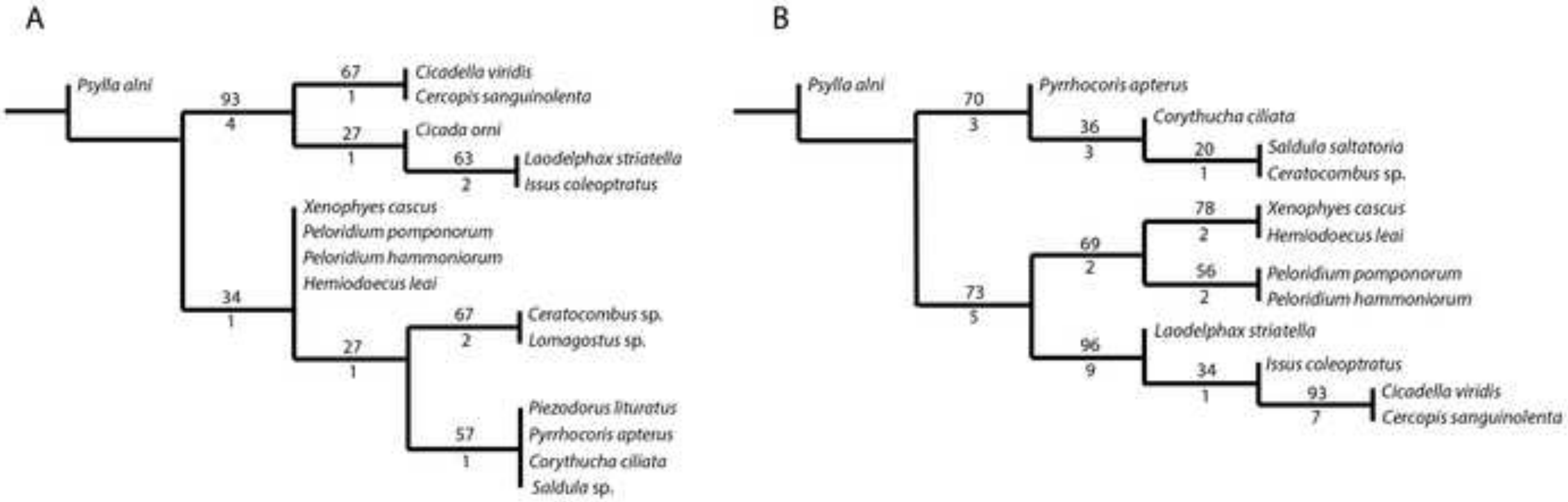

Click here to access/download
Supporting Information - Compressed/ZIP File Archive
Supplementary Files.zip
