# Supplementary material for: Moss bugs shed light on the evolution of complex bioacoustic systems
Source: PLoS One. 2024 Feb 23;19(2):e0298174. doi: 10.1371/journal.pone.0298174 (PMC10890781; doi:10.1371/journal.pone.0298174)
Supplement: Attachment — Submitted filename: Referee.docx [file pone.0298174.s009.docx]

**Referee’s comments to Manuscript Number PONE-D-23-39772**

The paper addresses the bioacoustic system in Hemiptera by analysing and comparing functional morphology, video films, and laser vibrometry from extant species of Peloridiidae (and ther fossil relatives) with other hemipteran bugs and thereby provide new insight in the origin of this important feature.

The used techniques are relatively easy to follow even for a reader with no personal experience in these methods, and the morphological characters are scored and used in phylogenetic analyses suggesting new evidence for the relationship between peloridiids and other hemipteran bugs, even though the support for such relationships is still inconclusive.

The paper is very well written, and I only have a few minor details that I would like to see changed.

1. It wonder why the newly generated morphological characters used for the phylogenetic analyses are not available and open for inspection and discussion.
2. While the paper reviews studies of the relationship between Peloridiids and other hemipteran bugs, the relationships within the family are not addressed, even though they could be used to discuss the difference between species from New Zealand (*Oiophysa*, *Xenophyes*, *Xenophysella*) on one side and species from Australia (*Hackeriella*, *Hemiodoecus*) and South America (*Peloridium*) on the other as outlined on lin 114-118. According to the recent study by Ye et al. (2019), *Peloridium* is sister group to all other peloridiids, and the fauna from New Zealand (and New Caledonia) is sister group to a clade of other South American taxa and another clade consisting of the species from Australia and Lord Howe Is.
3. On Fig. 1, it is quite confusing that two males of *Hackeriella weitchi* are shown on top of one another. It would make more sense to show a single male or a male and a female.
4. I wonder if the references for inferring the relationships between Peloridiidae and other hemipterans for Fig. 2 on line 83-86 are correct.

Other formalia:

- Do check if the right type of brackets are used (see. e.g. ref. 63 on l. 303-304.
- Reference 64 is first mentioned in line 350, which is after succeeding references, e.g. ref. 67 in line 346.
