# Supplementary material for: Moss bugs shed light on the evolution of complex bioacoustic systems
Source: PLoS One. 2024 Feb 23;19(2):e0298174. doi: 10.1371/journal.pone.0298174 (PMC10890781; doi:10.1371/journal.pone.0298174)
Supplement: Attachment — Submitted filename: Response to reviewers.docx [file pone.0298174.s010.docx]

“*Reviewers' comments:*

*Reviewer's Responses to Questions*

*Comments to the Author*

*1. Is the manuscript technically sound, and do the data support the conclusions?*

*The manuscript must describe a technically sound piece of scientific research with data that supports the conclusions. Experiments must have been conducted rigorously, with appropriate controls, replication, and sample sizes. The conclusions must be drawn appropriately based on the data presented.*

*Reviewer #1: Yes*

*Reviewer #2: Yes*

*2. Has the statistical analysis been performed appropriately and rigorously?*

*Reviewer #1: N/A*

*Reviewer #2: N/A*

*3. Have the authors made all data underlying the findings in their manuscript fully available?*

*The PLOS Data policy <http://www.plosone.org/static/policies.action#sharing> requires authors to make all data underlying the findings described in their manuscript fully available without restriction, with rare exception (please refer to the Data Availability Statement in the manuscript PDF file). The data should be provided as part of the manuscript or its supporting information, or deposited to a public repository. For example, in addition to summary statistics, the data points behind means, medians and variance measures should be available. If there are restrictions on publicly sharing data-e.g. participant privacy or use of data from a third party-those must be specified.*

*Reviewer #1: Yes*

*Reviewer #2: No*

*4. Is the manuscript presented in an intelligible fashion and written in standard English?*

*PLOS ONE does not copyedit accepted manuscripts, so the language in submitted articles must be clear, correct, and unambiguous. Any typographical or grammatical errors should be corrected at revision, so please note any specific errors here.*

*Reviewer #1: Yes*

*Reviewer #2: Yes*

*5. Review Comments to the Author*

*Please use the space provided to explain your answers to the questions above. You may also include additional comments for the author, including concerns about dual publication, research ethics, or publication ethics. (Please upload your review as an attachment if it exceeds 20,000 characters)*

***Reviewer #1****: The author is obviously a highly skilled morphologist and a very competent expert in the field of insect bioacoustics. The study addresses vibrational organs of a phylogenetically crucial taxon in the megadiverse Hemiptera.*”

We would like to thank the Editor of PLOS One and the reviewers for their careful review, and for the overwhelmingly positive assessment of our manuscript. We respond point-to-point in the sections below.

“*l. 23. I would avoid "derived Hemiptera", strictly speaking there are only derived characters/character states. I assume you mean Euhemiptera?*”

Yes, we mean Euhemiptera – this has now been amended (line 23).

“*l. 138: I recommend using Roman numerals for abdominal segments, tergites and sternites (like e.g. in Beutel et al. 2014) (e.g. tergite I).*”

We have now implemented this suggestion throughout the text (e.g. lines 104, 117, 137-138, 142, 144), and in all relevant illustrations (Figs. 3, 5-7, 10).

“*l. 149. I do not really like "in most Heteroptera" (or "most Auchenorrhyncha") but would rather use "most species/groups of...." Or "most heteropterans", but this is disputable.*”

We have now implemented your suggestion (line 148).

“*l. 180. Studies in prep. cannot be really cited, maybe use pers. comm. instead?*”

We now properly cite this statement (line 178).

“*There are some comparative statements in the morphological description (referring to Auchenorrhyncha) but in this specific case I have no objections.*”

Thank you for your comment. Indeed, the comparative statements are there in order to place said morphology into the necessary phylogenetic context.

“*Phylogenetic analysis*

*I mostly worked with maximum parsimony myself, but apparently Bayesian inference can produce better results, also concerning ancestral state reconstruction. But the use of MI is optional in this case.*”

Thank you for this comment. We also used maximum parsimony in this case, as it is one of the most broadly used cladistic methods and suitable for the hypotheses we wanted to test.

“*Literature: relevant studies are fully covered as far as I can see.*

*Language: very good*

*Illustrations: excellent*

*To summarize, this is an outstanding study, in terms of morphological documentation, vibration recording, phylogenetic evaluation, and evolutionary interpretation.*

*I recommend publication after (very) minor revision.*

*It was a pleasure to read this.*

*Rolf G. Beutel*”

We would like to thank Prof. Dr. Beutel for his helpful comments and very positive assessment of our manuscript.

**“*Reviewer #2:*** *The paper addresses the bioacoustic system in Hemiptera by analysing and comparing functional morphology, video films, and laser vibrometry from extant species of Peloridiidae (and ther fossil relatives) with other hemipteran bugs and thereby provide new insight in the origin of this important feature.*

*The used techniques are relatively easy to follow even for a reader with no personal experience in these methods, and the morphological characters are scored and used in phylogenetic analyses suggesting new evidence for the relationship between peloridiids and other hemipteran bugs, even though the support for such relationships is still inconclusive.*

*The paper is very well written, and I only have a few minor details that I would like to see changed.*”

We thank Reviewer 2 for their positive assessment of our work, and for their constructive feedback, which we address below.

“*1. It wonder why the newly generated morphological characters used for the phylogenetic analyses are not available and open for inspection and discussion.*”

The characters and the states as well as character matrices used for the TNT analyses are provided in the supplementary files 3A-C. We now provide an additional supplementary file with a list of all characters and detailed information on the states and the way those were coded. For more detailed information on morphological features please refer to reference [59].

“*2. While the paper reviews studies of the relationship between Peloridiids and other hemipteran bugs, the relationships within the family are not addressed, even though they could be used to discuss the difference between species from New Zealand (Oiophysa, Xenophyes, Xenophysella) on one side and species from Australia (Hackeriella, Hemiodoecus) and South America (Peloridium) on the other as outlined on lin 114-118. According to the recent study by Ye et al. (2019), Peloridium is sister group to all other peloridiids, and the fauna from New Zealand (and New Caledonia) is sister group to a clade of other South American taxa and another clade consisting of the species from Australia and Lord Howe Is.*”

Thank you for your comment. The intrafamiliar relationships of Peloridiidae are indeed interesting and they have been studied by the second co-author (Viktor Hartung) in his doctoral thesis [59]. His results agree with those by Ye et al. (2019) very closely - he obtained exactly the same intrafamiliar relationships. However, since the focus of our paper is quite different from [59] and we only use 5 Peloridiidae species in our present paper while [59] used 21, we do not think a discussion of the Peloridiidae intrafamiliar relationships would be very well supported with so few taxa.

“*3. On Fig. 1, it is quite confusing that two males of Hackeriella weitchi are shown on top of one another. It would make more sense to show a single male or a male and a female.*”

Thank you for your comment. Although we have a large collection of macrophotographs of live peloridiids, Fig. 1 used in this manuscript was the best in terms of resolution and clarity. This is one of the very few available photographs of living peloridiids, which are otherwise exclusively photographed once they have been euthanized. In most of the species that we examined (including the one imaged in Fig. 1), males will frequently mount each other, perhaps as part of a currently unstudied territorial behaviour. Fig. 1 therefore captures a natural behaviour of peloridiids.

“*4. I wonder if the references for inferring the relationships between Peloridiidae and other hemipterans for Fig. 2 on line 83-86 are correct.*”

These were incorrect – these citations have now been updated and corrected (lines 85-86).

“*Other formalia:*

** Do check if the right type of brackets are used (see. e.g. ref. 63 on l. 303-304.*”

I checked and corrected errors where they occur (e.g. line 299).

*“* Reference 64 is first mentioned in line 350, which is after succeeding references, e.g. ref. 67 in line 346.*”

Thank you for pointing this out. We have now corrected the order of citations (line 346).
